# Reliability of urinary charged metabolite concentrations in a large-scale cohort study using capillary electrophoresis-mass spectrometry

**DOI:** 10.1038/s41598-021-86600-9

**Published:** 2021-04-01

**Authors:** Yoshiki Ishibashi, Sei Harada, Ayano Takeuchi, Miho Iida, Ayako Kurihara, Suzuka Kato, Kazuyo Kuwabara, Aya Hirata, Takuma Shibuki, Tomonori Okamura, Daisuke Sugiyama, Asako Sato, Kaori Amano, Akiyoshi Hirayama, Masahiro Sugimoto, Tomoyoshi Soga, Masaru Tomita, Toru Takebayashi

**Affiliations:** 1 Department of Preventive Medicine and Public Health, Keio University School of Medicine, 35 Shinanomachi, Shinjuku, Tokyo Japan; 2 Institute for Advanced Biosciences, Keio University, Tsuruoka, Yamagata Japan; 3 Faculty of Nursing And Medical Care, Keio University, Fujisawa, Kanagawa Japan; 4 Faculty of Environment and Information Studies, Keio University, Fujisawa, Kanagawa Japan

**Keywords:** Molecular biology, Biomarkers, Molecular medicine, Medical research, Epidemiology

## Abstract

Currently, large-scale cohort studies for metabolome analysis have been launched globally. However, only a few studies have evaluated the reliability of urinary metabolome analysis. This study aimed to establish the reliability of urinary metabolomic profiling in cohort studies. In the Tsuruoka Metabolomics Cohort Study, 123 charged metabolites were identified and routinely quantified using capillary electrophoresis-mass spectrometry (CE-MS). We evaluated approximately 750 quality control (QC) samples and 6,720 participants’ spot urine samples. We calculated inter- and intra-batch coefficients of variation in the QC and participant samples and technical intraclass correlation coefficients (ICC). A correlation of metabolite concentrations between spot and 24-h urine samples obtained from 32 sub-cohort participants was also evaluated. The coefficient of variation (CV) was less than 20% for 87 metabolites (70.7%) and 20–30% for 19 metabolites (15.4%) in the QC samples. There was less than 20% inter-batch CV for 106 metabolites (86.2%). Most urinary metabolites would have reliability for measurement. The 96 metabolites (78.0%) was above 0.75 for the estimated ICC, and those might be useful for epidemiological analysis. Among individuals, the Pearson correlation coefficient of 24-h and spot urine was more than 70% for 59 of the 99 metabolites. These results show that the profiling of charged metabolites using CE-MS in morning spot human urine is suitable for epidemiological metabolomics studies.

## Introduction

The use of large-scale metabolomics for prospective epidemiological studies is becoming more common, and various metabolomics epidemiological studies, such as Cooperative Health Research in the Region of Augsburg (KORA), Twins UK registry, and American cohorts such as the Framingham Heart Study (FHS) Offspring, are being conducted^1–6^. Such large-scale metabolomics studies may allow for the prediction of chronic diseases such as Alzheimer's disease^7^, cardiovascular disease^8^, and chronic kidney disease (CKD)^9^. Thus, we initiated the Tsuruoka Metabolomics Cohort Study (TMCS) in Japan and have enrolled 11,002 participants since April 2012^10–14^. In this study, capillary electrophoresis–mass spectrometry (CE-MS) was used to assess charged metabolites and liquid chromatography-mass spectrometry (LC–MS) for lipid metabolites.

In metabolomics studies, the use of urine as a sample has several advantages such as its ready availability, ease of obtaining, and lower complexity than other body fluids such as blood^15,16^. Metabolomic studies generally need to report reliability in measurement, as measurement accuracy is more variable than in routine tests^17–19^. We have already reported the reliability and accuracy of plasma metabolites in cohort studies through another study conducted by us^13^. Previous studies have investigated the measurement reliability for urinary iodine^20^ or urinary hydroxypyrene glucuronide^21^ by CE-MS. However, there are few studies which comprehensively evaluate validation for urinary metabolites in large-scale epidemiological studies. There are also studies which have applied CE-MS in large-scale urine analyses (n > 2000) , such as peptides relevant to cancer diagnosis^22^, but there are few studies that have examined the validity of urinary metabolites in a cohort of general population.

Thus, in this study, we aimed to examine the reliability of large-scale urinary metabolomic profiling, assessed with CE-MS platform, using approximately 750 quality control (QC) samples and 6,720 participants’ spot urine samples over a 56-month baseline period in a population-based cohort study. We also compared the concentration of the metabolites in spot urine samples with that obtained in 24-h collected urine samples for reference, using a sub-cohort.

## Materials and methods

### Study population and sample collection

**Figure 1:**
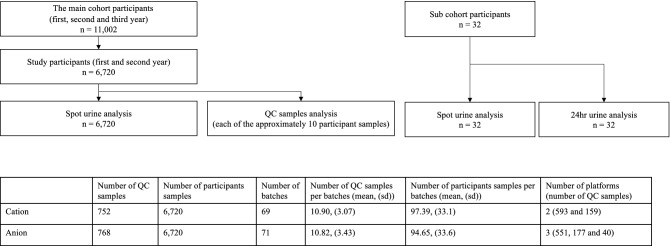
Overview of Study design.

TMCS is a Japanese cohort study, initiated in April 2012 (Tsuruoka City, Yamagata Prefecture, Japan), involving 11,002 participants aged 35 to 74 years^10–14^. The participants were attendees of the annual municipal or workplace health checkup programs held at four sites in the city at baseline (April 2012–March 2015). The study design is illustrated in Fig. 1.

TMCS was particularly designed to discover metabolomics biomarkers for common diseases and disorders related to environmental and genetic factors. All participants completed a comprehensive questionnaire on lifestyle, dietary habits, and medical history. Biological samples including serum, plasma, urine, and deoxyribonucleic acid (DNA) were collected, and medical examination data recorded during the health checkup programs were also collected at recruitment. Information on alcohol consumption and smoking habits, dietary pattern, stress, and level of physical activity was collected through a standardized self-management questionnaire, and these data were verified in person. The procedures for recruitments of the TMCS have also been described in detail in previous studies^10–14^. To avoid variation due to fasting state and circadian rhythm, urine samples were collected from each participant in the morning between 8:30 am and 10:30 am after an overnight fast.

QC samples were prepared by mixing approximately 10 randomly selected participant samples, and analyzed every 10 runs in each batch to evaluate analytical validation. Finally, the QC samples were measured 752 times in 69 batches for cation analysis, and 768 times in 71 batches for anion analysis. Baseline participant samples were analyzed to evaluate the variance of participants’ metabolites in the population. There are 2 CE-TOF platforms in cation and 3 of those in anion. A total of 6,720 samples from included participants were analyzed from the TMCS baseline cohort: first and second year (April 2012–March 2014). Follow-up participants were not included in these samples.

To compare the metabolite concentrations in the spot urine samples with that in the 24-h collected urine samples as a reference, a sub-cohort consisting of 32 TMCS participants within the cohort was set in 2013. These participants also answered a questionnaire on lifestyle, dietary habits, and medical history. In the sub-cohort analysis, boric acid was added to the samples during pretreatment as antioxidant.

### Sample preprocessing, instruments and analytical conditions

The samples were initially vortexed for 30 s, followed by centrifugation at 2,300 × *g* for 5 min at 4 °C. They were then diluted according to the creatinine concentration (Table S1) with Milli-Q water and Milli-Q water containing internal standards (2 mM each of methionine sulfone and camphor-10-sulfonic acid). We found that a creatinine concentration of < 10 mg/dl does not cause ion saturation in the mass spectrometer. Hence, we set this as the upper limit for diluting urine. Preparation of urine samples was performed manually.

Mass spectrometry-based metabolomic profiling was performed with fasting urine samples using capillary electrophoresistime-of-flight mass spectrometry (CE-TOFMS). CE-TOFMS analysis of cationic and anionic metabolites was performed as described previously^23–25^. Briefly, cationic metabolites were separated on a fused-silica capillary column (50 μm i.d. × 100 cm total length) filled with 1 M formic acid as the electrolyte, and a methanol/water (50%, v/v) containing 0.01 μM hexakis(2,2-difluoroethoxy)phosphazene (Hexakis) was delivered as a sheath liquid at a rate of 10 μL/min. The capillary temperature was maintained at 20 °C. The sample solution was injected at 5 kPa for 5 s, and a positive voltage of 30 kV was applied. ESI-TOFMS was conducted in the positive ion mode, and the capillary, fragmentor, skimmer, and Oct RF voltages were set at 4,000, 75, 50, and 500 V, respectively. The nebulizer gas pressure was configured at 7 psig and the heated nitrogen gas (300 °C) was supplied at a rate of 10 l/min. Anionic metabolites were separated using a commercially available COSMO( +) capillary (50 μm i.d. × 105 cm, Nacalai Tesque, Kyoto, Japan) filled with 50 mM ammonium acetate (pH 8.5) as the electrolyte, and ammonium acetate (5 mM) in 50% (v/v) methanol/water containing 0.01 μM Hexakis was delivered as sheath liquid at a rate of 10 μL/min. The sample solution was injected at 5 kPa for 30 s, and a negative voltage of 30 kV was applied. ESI-TOFMS was conducted in the negative ion mode, and the capillary, fragmentor, skimmer, and Oct RF voltages were set at 3,500, 100, 50, and 500 V, respectively. Other conditions were identical for the cationic metabolite analysis. In both modes, the automatic recalibration function was used to correct the analytical variation of exact masses for each run as described previously^25^. Mass spectra were acquired at a rate of 1.5 cycles/s over a 50–1,000 m*/z* range. We performed non-targeted analysis of urine QC samples with CE-MS. Among these, we investigated whether standard reagents were available for the compounds whose peaks could be identified from QC samples, and targeted the available compounds for analysis.

In order to keep the state of the instrument constant, we set the number of measurement samples in one batch less than 100, and replaced the capillaries and wash the MS ion source for each batch. Mass calibration was performed immediately before the measurement of each batch, and mass tuning was performed once a month.

### Statistical analysis

Since missing values were created by being below the measurement limit, half of the lowest detected values were imputed for metabolites that were not detected^26^. As we performed previously^13^, inter- and intra-batch variance for each metabolite concentration in the QC samples was calculated to evaluate the reproducibility of the data. To control the effects of the batch, a linear mixed model was formulated, as shown in Eq. (1).

1 $$Y_{i} = \, \mu \, + \, B_{i} + \, \varepsilon_{i}$$

The observed metabolite concentration (Y), inter—and intra-batch variance for each metabolite (μ), random effects common to each batch (B), and residual error (ε) are defined in the formula. We calculated the coefficient of variation (CV) by dividing the standard deviation as estimated from this model by the mean. Pearson correlation coefficients between the inter- and intra-batch CV were then calculated. These analyses were also conducted with participant samples to assess inter- and intra-batch variance. The intraclass correlation coefficient (ICC) was calculated to assess the reliability of the metabolite concentrations^27,28^. This value was calculated from the variance of the measurement errors and the total variance,

2 $${\text{ICC }} = \, 1 - \, \sigma_{E}^{2} /\sigma_{T}^{2}$$

where σ_E_^2^ is the variance of the measurement errors and σ_T_^2^ is the total variance, as shown in Eq. (2). Although we could not calculate the ICC for participant samples as there were no replicates, we calculated technical errors from a large number of replicates for QC samples considered to be representative of the population samples. We made an approximate calculation of ICC, substituting the CV of QC samples for error variance and CV of participant samples for the total variance, as shown in Eq. (3).

3 $${\text{Approximate}}\;{\text{ICC }} = \, 1 - \left( {{\text{CV}}\;{\text{QC}}} \right)^{2} / \, \left( {{\text{CV}}\;{\text{participant}}} \right)^{2}$$

When creatinine correction was performed, it is well known that substances tend to be lower in concentrated urine samples than in diluted urine samples. Second, some diseases and medications can cause fluctuations in urine creatinine levels. Therefore, a sensitivity analysis was conducted excluding samples with creatinine values > 3.0 g/g/L or < 0.3 g / g/L from participants^29^.

For sub-cohort analysis, metabolites in 24-h urine samples and spot urine samples were compared among individuals, and Pearson's correlation coefficient was calculated for each individual. To account for major factors that may affect urinary metabolite concentrations, a regression analysis was performed, and the slope was compared between the spot and 24-h urine samples. The explanatory variables included age, sex, alcohol consumption, and smoking, all of which are known to affect metabolite concentrations^10,30,31^.

Statistical analyses were performed using R version 3.5.2 (2018–12-20) (R Core Team 2018, 2018, The R Foundation for Statistical Computing, Vienna, Austria).

### Ethical approval

This study was approved by the Medical Ethics Committee of the School of Medicine, Keio University, Tokyo, Japan (Approval No 20110264 and No 20130207 for the entire cohort study and the sub-cohort one, respectively). Informed consent was obtained in written form from all the participants included in the studies. All research was performed in accordance with the relevant guidelines and regulations.

## Results

### CV for QC samples

**Figure 2:**
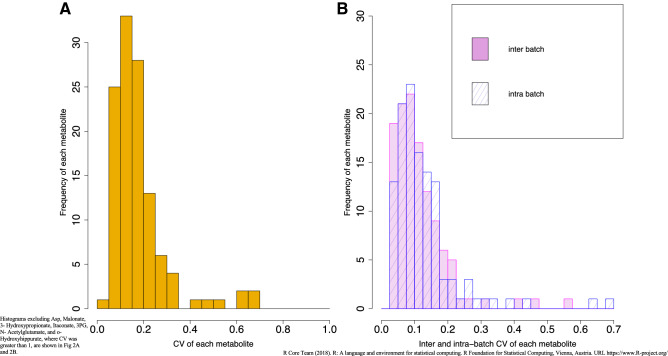
Histogram of CV for each metabolite in QC samples. (**A**) Total CV of each metabolite. (**B**) Inter and Intra-batch CV of each metabolite.

123 charged urinary metabolites were identified and routinely quantified in this study. Among these metabolites, CV was less than 20% for 87 metabolites (70.7%), 20%–30% for 19 metabolites (15.4%), and more than 30% for 17 (13.8%) metabolites (Fig. 2A). The median CV was 11.7% and 17.8% for the cation and anion compounds, respectively. Table S2 shows the CV values of 123 metabolite concentrations in the QC samples. Inter-batch CV was ˂ 20% for 106 compounds (86.2%). Intra-batch CV was ˂ 20% for 103 compounds (83.7%) (Fig. 2B). There were similar values between inter- and intra-batch CVs (medians, 6.9% and 9.0% for cations; 13.1% and 11.7% for anions), and they were also strongly correlated (Pearson’s r = 0.89, N = 123, *P* < 0.001) (Fig. S1).

### CV in participant samples

**Figure 3:**
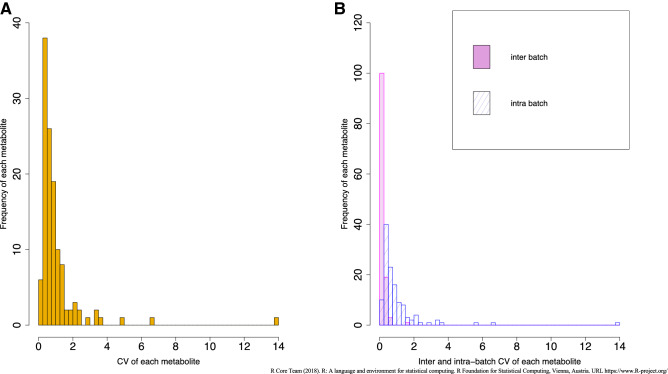
Histogram of CV for each metabolite in participants samples. (**A**) Total CV of each metabolite. (**B**) Inter and Intra-batch CV of each metabolite.

**Figure 4:**
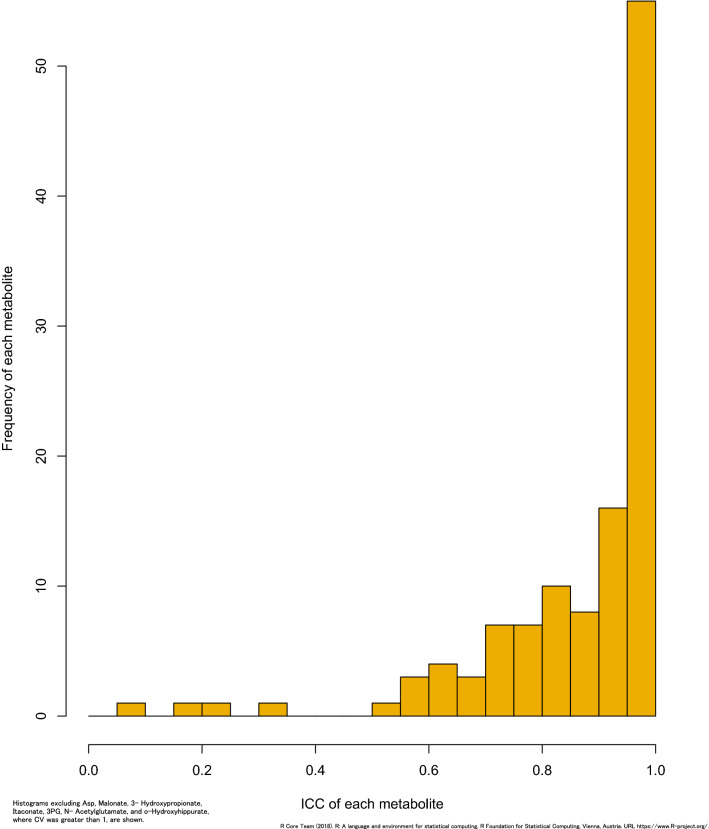
The distribution of the estimated ICC for each metabolite.

Figure 3A and B show the distribution of the total, inter-, and intra-batch CVs among participants. The medians of total, inter-, and intra-batch CVs were 81.3%, 15.6%, and 79.9% for cations, respectively, and 61.9%, 19.6%, and 55.5% for anions, respectively. Predictably, the CV values of the participant samples were larger than those of the QC samples. There was a larger intra-batch CV than inter-batch CV in participant samples, in contrast to QC samples. Table S2 shows a statistical summary of 123 metabolite concentrations quantified in participant samples. Figure 4 shows the distribution of the estimated ICC. The estimated ICC was > 0.75 for 96 metabolites (78.0%), 0.40.0.75 for 18 metabolites (14.6%), and < 0.40 for 9 metabolites (7.3%) . The estimated ICC values are shown in Table S2(metabolites of ICC < 0 are excluded in Fig. 4). The results of the sensitivity analysis by exclusion based on the creatinine levels were similar to that seen in the overall participants (Table S3).

### Comparison of spot and 24-h urine samples

**Figure 5:**
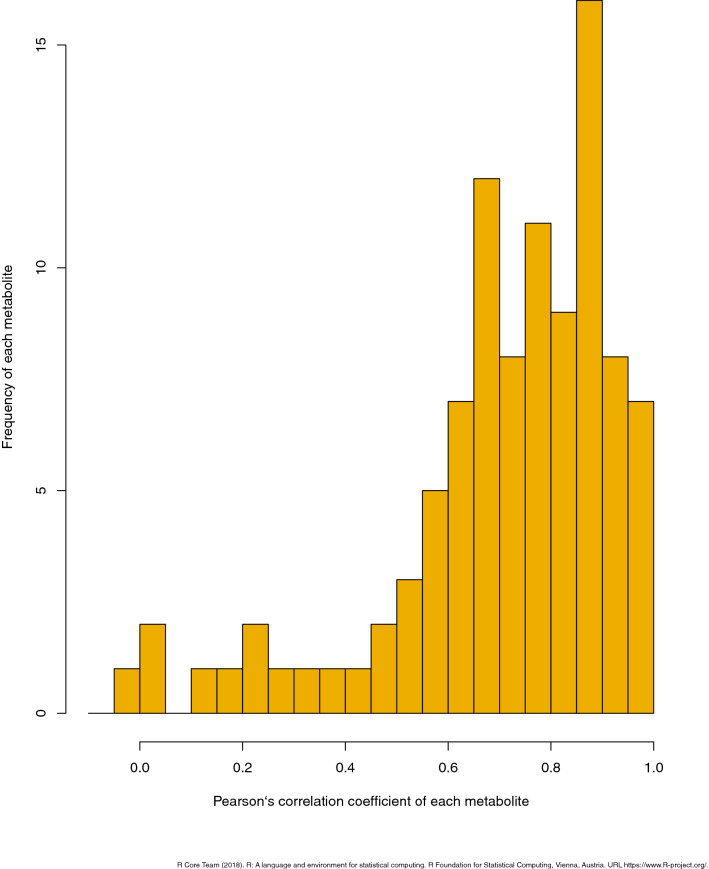
Pearson’s correlation coefficient for 24-h and spot morning urine samples.

Among individuals, the Pearson’s correlation coefficient for 24-h and spot morning urine samples was > 0.7 for 59 (59.6%) of the 99 metabolites (Fig. 5). The medians of the Pearson’s correlation coefficients for 24-h and spot morning urine samples were 0.75 and 0.80 for cations and anions, respectively. The association between 24-h urine and spot urine samples was strong for the majority of the metabolites. A statistical summary of metabolites measured in the spot and 24-h samples is shown in Table S4. The total CV distribution among metabolites is shown in Fig. S2. The medians of the total CV for the spot and 24-h urine samples were 37.0% and 41.9% for cations and 40.1% and 41.9% for anions, respectively. The regression coefficients of the basic demographic characteristics (age, sex, alcohol consumption, and smoking) were comparable between 24-h urine and spot urine samples (Table S5) for most of the metabolites.

## Discussion

In this large-scale epidemiological study, the reproducibility of 123 charged compounds in urine samples as quantified by CE-MS was reported to be good to high: QC CV for 106 compounds (86% of all) was less than 30%, while the measurement period was 56 months. Inter-batch CV for QC samples was less than 20% for 106 compounds (86.2%). A high CV was observed among the participants, and was caused by intra-batch CV rather than inter-batch CV. Therefore, the impact of inter- and intra-individual variability is likely to be greater than the impact of each measurement batch. In general, inter-batch CV values are often higher than the intra-batch values. However, as shown in our previous study^13^, intra-batch CV was higher than inter-batch CV in the participants. This is evidence of the diversity of metabolites among the participants, and the same phenomenon has been observed in urine. Metabolomic cohort studies have been used to measure urinary metabolites^32,33^, but the reliability of metabolite assessments and the variance between each batch and all of the participants have yet to be reported in most studies. In previous metabolomics studies, QC sample methods have been widely used to evaluate reproducibility^34^. Plasma QC CV, which has been reported previously, is lower than that of urine^13^. However, features with a QC CV < 20% are often considered to have good reproducibility, as recommended by the US Food and Drug Administration^35^. A QC CV < 30% is also considered acceptable^34,36^.

This is the first study to report inter- and intra-batch CVs in urine samples from a cohort study. Reducing inter-batch variability is an important issue in large-scale metabolomics studies^13,36,37^. The results showed that a metabolomics assay of cations and anions effectively controlled the batch-to-batch effects of many measured compounds in this study. Our estimated ICC was greater than 0.40 for most measured metabolites, except for nine (N-acetylneuraminate, 2-oxoglutarate, cysteine S-sulfate, o-hydroxyhippurate, 4-hydroxy-3-methoxymandelate, 3-hydroxypropionate, 3-phosphoglycerate, malonate, and itaconate). Metabolites with a low reproducibility but an ICC > 0.40 may be of value as biomarkers, provided careful evaluation of their measurement errors is done^13^. With sufficient large ICCs for metabolites (it means QC CV values are much less than participants’ CV), epidemiological analysis of these metabolites becomes possible. And also, it is important to calculate the CV of the population (intra-individual variation + inter-individual variation + methodological variation including measurement error) epidemiological use of urinary metabolites^38^. The metabolites with low ICCs didn’t belong to the same class or have low concentrations. It may be possible to obtain good ICCs for these metabolites in the future by changing the analytical conditions.

The metabolite concentrations in spot urine samples following overnight fasting conditions and 24-h urine samples were comparable in majority of the metabolome, even after considering the basic demographic variables. Since 24-h urine samples are known to be stable and aid in the quantification of metabolites^39,40^, early morning fasting spot urine metabolomic measurements can be used as a surrogate index for 24-h urine measurements; thus, they are suitable for large-scale biomarker studies.

Although recent studies have used blood metabolites to detect many diseases^1–6^, studies using urinary metabolites are also on the rise^15,16^ due to the ease of availability and collection of samples as well as its low complexity. In a nuclear magnetic resonance (NMR)-based metabolomics cohort study, CVs of 43 urinary metabolites in QC samples were reported to be low^17^; however, CE-MS allows us to quantify a larger number of metabolites at lower concentrations compared to NMR. In a cohort, the accuracy of measurement of metabolites is important for personalized medicine and disease onset prediction because the ability to measure a larger number of metabolites will increase the chance to explore new biomarkers for various diseases. For example, metabolites such as symmetric dimethylarginine, asymmetric dimethylarginine, and ethanolamine, which were detected using CE-MS in this study but not in NMR, not only had low CVs but also showed similar relationships between spot urine and 24-h urine samples across the various groups. This indicates that these spot urine metabolites may be useful in predicting CKD and rheumatic disease, which are conditions where biomarkers such as these have already been reported^41,42^.

This study had some limitations. The CVs of some metabolites (17 metabolites) were more than 30% in the QC samples. Thus, further improvement in the quality of measurement is required for these metabolites. Second, this population can be considered representative of the Japanese population with a homogenous genetic background; however, the diversity of environmental factors must also be taken into account. Therefore, further research is needed in Japan, and in other countries, to enable generalization of the findings. Although international comparative studies have been conducted in plasma samples, further studies with urine samples are needed. Third, in the future study, robustness check would be considered using alternative methods for normalization/adjustment to hydration status besides creatinine, such as osmolality^43^ given the known limitations of creatinine (e.g., sex and age-dependent, as well as dependent on protein intake and normal kidney function). Fourth, a stability study in order to examine the robustness of urinary metabolites for clinical or epidemiology studies would be worth as further study^44^. At last, though we investigated whether standard reagents were available for the compounds whose peaks could be identified from QC samples, and targeted the available compounds for analysis, even if there is a specific metabolite that can be detected only in a specific disease, it cannot be detected by this method. However, since the data was acquired in scan mode, it can be re-analyzed in the future analysis.

In conclusion, this study showed that the CE-MS platform provides reliable values for urine metabolites, as assessed in a large-scale cohort study. CE-MS provides high-quality metabolomics data to help us understand the relationship between metabolites and disease risk.

## Supplementary Information

Supplementary Information

## Data Availability

The most relevant data are within the paper. Raw data cannot be made publicly available, as study participants did not consent to have their information freely accessible. Based on these consents, the Ethics Committee for Tsuruoka Metabolomics Cohort Study inhibits any public data sharing because data contain potentially identifying or sensitive disease information. This committee includes representatives of Tsuruoka citizens, the administration of Tsuruoka City, a lawyer, and expert advisers. Data accession requests may be sent to the administration of the Ethics Committee for Tsuruoka Metabolomics Cohort Study. The data will be shared after a review of the purpose and with permission from the ethics committee. Data requests can be made to Sei Harada, seiharada@keio.jp. The source code and analysis generated during the current study are not publicly available, but are available from the corresponding author upon reasonable request.
