# Supplementary material for: Reliability of urinary charged metabolite concentrations in a large-scale cohort study using capillary electrophoresis-mass spectrometry
Source: Sci Rep. 2021 Apr 1;11:7407. doi: 10.1038/s41598-021-86600-9 (PMC8016858; doi:10.1038/s41598-021-86600-9)
Supplement: Supplementary file 1 — Supplementary Information [file 41598_2021_86600_MOESM1_ESM.pdf]

Reliability of urinary charged metabolite concentrations in a large-scale cohort study using capillary electrophoresis-mass spectrometry

Yoshiki Ishibashi[1], Sei Harada[1,2], Ayano Takeuchi[1], Miho Iida[1], Ayako Kurihara[1], Suzuka Kato[1], Kazuyo Kuwabara[1], Aya Hirata[1], Takuma Shibuki[1], Tomonori Okamura[1], Daisuke Sugiyama[1,3], Asako Sato[2], Kaori Amano[2], Akiyoshi Hirayama[2], Masahiro Sugimoto[2], Tomoyoshi Soga,[2,4] Masaru Tomita[2,4], Toru Takebayashi[1,2,*]

Affiliation

[1]Department of Preventive Medicine and Public Health, Keio University School of Medicine, Tokyo, Japan

[2]Institute for Advanced Biosciences, Keio University, Tsuruoka, Yamagata, Japan

[3]Faculty of Nursing And Medical Care, Keio University, Fujisawa, Kanagawa, Japan

[4]Faculty of Environment and Information Studies, Keio University, Fujisawa, Kanagawa, Japan

*Corresponding author:

Toru Takebayashi, MD, PhD

Department of Preventive Medicine and Public Health, Keio University School of Medicine,

35 Shinanomachi, Shinjuku, Tokyo, Japan

Tel: +81- 5363-3758; Fax: +81-3- 3359-3686

E-mail: ttakebayashi@a3.keio.jp

Table S1. Urine dulation protocol.

| Creatinine concentration | Dilution rate | Urine | 2m M IS 1,2§1§2 and Milli-Q | Milli-Q |
|---|---|---|---|---|
| 100mg/dl ~ | 20-fold | 10μL | 20μL | 170μL |
| 50~99mg/dl | 10-fold | 20μL | 20μL | 160μL |
| 25~49mg/dl | 5-fold | 40μL | 20μL | 140μL |
| 10-24mg/dl | 2-fold | 100μL | 20μL | 80μL |
| ~9mg/dl | 1.11-fold | 180μL | 20μL | 0μL |

**§1 IS1**: L-Methionine sulfone (Wako 502-76641), and CSA (Wako 037-01032) MES (Dojindo 349-01623).

**§2 IS2**: 3-Aminopyrrolidine (Aldrich 404624) and Trimesate (Wako 206-03641)

**Table S2. Statistical summary of measured metabolites.**

| Metabolite name (HMDB ID) | QC samples | | | Participant samples | | | | | | | | | | | Estimated ICC* | Metabolite name |
|---|---|---|---|---|---|---|---|---|---|---|---|---|---|---|---|---|
| | Total CV | Inter-batch CV | Intra-batch CV | Total CV | Inter-batch CV | Intra-batch CV | Mean (μmol/L) | SD | 1st quartile | Median | 3rd Quartile | Number of ND | % of ND | Lowest detection value | | |
| **Cations** | | | | | | | | | | | | | | | | Cations |
| Gly (HMDB0000123) | 5.5% | 4.1% | 3.7% | 69.0% | 21.1% | 65.9% | 75.33 | 52.00 | 42.08 | 61.02 | 91.36 | 0 | 0.00% | 5.11 | 0.993568754 | Gly |
| Trimethylamine N-oxide (HMDB0000925) | 11.0% | 9.1% | 5.8% | 115.0% | 30.2% | 111.1% | 63.96 | 73.52 | 21.47 | 36.25 | 77.10 | 19 | 0.28% | 0.11 | 0.990878145 | Trimethylamine N-oxide |
| beta-Ala (HMDB0000056) | 18.1% | 6.9% | 16.7% | 91.4% | 15.5% | 90.1% | 1.26 | 1.15 | 0.66 | 1.14 | 1.69 | 1080 | 16.07% | 0.15 | 0.960844097 | beta-Ala |
| Ala (HMDB0000161) | 5.8% | 3.9% | 4.3% | 69.7% | 14.2% | 68.3% | 21.07 | 14.69 | 13.11 | 17.95 | 25.37 | 0 | 0.00% | 3.26 | 0.993058647 | Ala |
| 3-Aminoisobutyrate (HMDB0003911) | 6.6% | 5.1% | 4.1% | 114.2% | 11.5% | 113.7% | 49.84 | 56.94 | 6.20 | 18.98 | 95.51 | 0 | 0.00% | 0.43 | 0.996656468 | 3-Aminoisobutyrate |
| 2AB (HMDB0000452) | 19.6% | 7.8% | 17.9% | 83.0% | 14.0% | 81.8% | 1.10 | 0.91 | 0.75 | 1.02 | 1.35 | 292 | 4.35% | 0.20 | 0.944219987 | 2AB |
| N,N-Dimethylglycine (HMDB0000092) | 9.5% | 5.4% | 7.7% | 326.8% | 17.3% | 326.4% | 6.91 | 22.58 | 3.62 | 5.31 | 7.72 | 13 | 0.19% | 0.34 | 0.999162255 | N,N-Dimethylglycine |
| Choline (HMDB0000097) | 12.9% | 10.6% | 7.5% | 49.7% | 15.0% | 47.4% | 3.50 | 1.74 | 2.46 | 3.20 | 4.10 | 0 | 0.00% | 0.85 | 0.932398936 | Choline |
| Ser (HMDB0000187) | 5.9% | 4.2% | 4.1% | 54.7% | 11.8% | 53.5% | 32.89 | 18.00 | 23.04 | 29.48 | 38.71 | 34 | 0.51% | 0.40 | 0.988286813 | Ser |
| Pro (HMDB0000162) | 17.8% | 7.8% | 16.0% | 142.6% | 19.7% | 141.3% | 0.93 | 1.33 | 0.55 | 0.75 | 1.03 | 487 | 7.25% | 0.15 | 0.984391516 | Pro |
| Guanidinoacetate (HMDB0000128) | 6.5% | 4.9% | 4.1% | 50.0% | 16.1% | 47.5% | 59.20 | 29.62 | 37.22 | 54.36 | 75.97 | 0 | 0.00% | 2.41 | 0.983266856 | Guanidinoacetate |
| Val (HMDB0000883) | 15.8% | 7.9% | 13.7% | 286.7% | 14.1% | 286.3% | 4.26 | 12.22 | 3.24 | 3.99 | 4.82 | 109 | 1.62% | 0.26 | 0.996976515 | Val |
| Betaine (HMDB0000043) | 11.2% | 6.3% | 9.2% | 148.7% | 22.4% | 147.1% | 19.94 | 29.64 | 8.72 | 12.70 | 20.21 | 21 | 0.31% | 1.30 | 0.99435097 | Betaine |
| Thr (HMDB0000167) | 4.9% | 2.8% | 4.0% | 95.4% | 17.5% | 93.8% | 15.58 | 14.86 | 9.23 | 12.68 | 18.03 | 2 | 0.03% | 1.31 | 0.997346579 | Thr |
| Taurine (HMDB0000251) | 11.5% | 10.2% | 5.5% | 70.1% | 15.7% | 68.3% | 172.27 | 120.76 | 96.35 | 150.23 | 216.96 | 5 | 0.07% | 5.85 | 0.973256821 | Taurine |
| Creatine (HMDB0000064) | 5.2% | 3.7% | 3.7% | 153.8% | 41.3% | 148.1% | 72.95 | 112.18 | 7.73 | 20.58 | 93.06 | 0 | 0.00% | 1.70 | 0.998854809 | Creatine |
| Ile (HMDB0000172) | 22.7% | 15.0% | 17.1% | 1384.6% | 64.3% | 1383.1% | 1.52 | 21.01 | 0.94 | 1.18 | 1.47 | 47 | 0.70% | 0.06 | 0.999731415 | Ile |
| Leu (HMDB0000687) | 7.6% | 3.9% | 6.6% | 365.5% | 13.0% | 365.3% | 3.49 | 12.77 | 2.55 | 3.14 | 3.87 | 37 | 0.55% | 0.10 | 0.99956267 | Leu |
| Asn (HMDB0000168) | 9.2% | 4.4% | 8.1% | 81.3% | 15.0% | 79.9% | 11.92 | 9.69 | 7.84 | 10.25 | 13.80 | 14 | 0.21% | 1.79 | 0.987165928 | Asn |
| Ornithine (HMDB0000214) | 14.5% | 6.9% | 12.8% | 178.7% | 19.5% | 177.7% | 1.94 | 3.46 | 1.16 | 1.51 | 2.04 | 40 | 0.60% | 0.33 | 0.993393897 | Ornithine |
| Asp (HMDB0000191) | 137.1% | 84.4% | 107.8% | 334.8% | 50.8% | 330.9% | 0.30 | 1.02 | 0.10 | 0.10 | 0.10 | 5529 | 82.28% | 0.19 | 0.832306516 | Asp |
| Hypoxanthine (HMDB0000157) | 6.9% | 4.8% | 5.0% | 49.8% | 17.6% | 46.6% | 13.07 | 6.50 | 8.88 | 12.28 | 16.18 | 2 | 0.03% | 1.03 | 0.980688346 | Hypoxanthine |
| 1-Methylnicotinamide (HMDB0000699) | 16.3% | 12.8% | 9.0% | 46.6% | 16.9% | 43.3% | 10.40 | 4.85 | 6.97 | 9.71 | 12.98 | 0 | 0.00% | 0.65 | 0.878446857 | 1-Methylnicotinamide |
| Trigonelline (HMDB0000875) | 11.6% | 6.6% | 9.5% | 93.3% | 20.1% | 91.2% | 16.16 | 15.08 | 6.56 | 11.37 | 20.50 | 1 | 0.01% | 0.51 | 0.984671736 | Trigonelline |
| Urocanate (HMDB0000301) | 26.7% | 17.8% | 20.6% | 221.8% | 16.0% | 221.2% | 2.34 | 5.20 | 1.63 | 2.26 | 2.96 | 781 | 11.62% | 0.28 | 0.985464852 | Urocanate |
| Proline betaine (HMDB0004827) | 11.7% | 6.5% | 9.7% | 146.5% | 32.6% | 142.9% | 15.20 | 22.27 | 3.62 | 6.80 | 16.95 | 4 | 0.06% | 0.55 | 0.993590601 | Proline betaine |
| gamma-Butyrobetaine (HMDB0001161) | 21.3% | 8.6% | 19.5% | 112.6% | 18.2% | 111.2% | 0.36 | 0.40 | 0.09 | 0.27 | 0.48 | 1569 | 23.35% | 0.04 | 0.96417581 | gamma-Butyrobetaine |
| Gln (HMDB0000641) | 6.0% | 4.5% | 3.8% | 84.4% | 15.1% | 83.1% | 60.94 | 51.41 | 41.80 | 55.16 | 72.90 | 2 | 0.03% | 5.67 | 0.994939379 | Gln |
| Lys (HMDB0000182) | 5.5% | 3.7% | 4.0% | 141.3% | 18.3% | 140.1% | 40.80 | 57.64 | 15.20 | 26.16 | 46.98 | 0 | 0.00% | 2.52 | 0.998494161 | Lys |
| Glu (HMDB0000148) | 15.8% | 7.4% | 13.9% | 65.5% | 13.3% | 64.1% | 2.30 | 1.51 | 1.61 | 2.16 | 2.78 | 165 | 2.46% | 0.37 | 0.942011104 | Glu |
| Met (HMDB0000696) | 25.9% | 8.9% | 24.3% | 178.9% | 13.2% | 178.4% | 1.46 | 2.60 | 1.07 | 1.36 | 1.71 | 230 | 3.42% | 0.28 | 0.979054536 | Met |
| His (HMDB0000177) | 6.5% | 5.3% | 4.0% | 55.4% | 10.2% | 54.5% | 85.95 | 47.59 | 54.05 | 76.99 | 107.97 | 0 | 0.00% | 2.56 | 0.986012119 | His |
| alpha-Aminoadipate (HMDB0000510) | 8.3% | 3.8% | 7.4% | 119.9% | 14.0% | 118.9% | 4.61 | 5.53 | 3.19 | 4.23 | 5.50 | 10 | 0.15% | 0.40 | 0.995207449 | alpha-Aminoadipate |
| Carnitine (HMDB0000062) | 12.1% | 7.7% | 9.3% | 94.6% | 18.1% | 93.0% | 18.30 | 17.32 | 5.59 | 13.22 | 25.81 | 0 | 0.00% | 0.32 | 0.983704556 | Carnitine |
| Phe (HMDB0000159) | 6.9% | 4.3% | 5.3% | 96.3% | 10.4% | 95.7% | 6.89 | 6.64 | 5.08 | 6.37 | 8.07 | 0 | 0.00% | 0.63 | 0.994843568 | Phe |
| 3-Methylhistidine (HMDB0000479) | 5.3% | 3.9% | 3.7% | 88.7% | 12.2% | 87.9% | 81.06 | 71.89 | 38.30 | 55.64 | 96.85 | 0 | 0.00% | 12.69 | 0.996419203 | 3-Methylhistidine |
| Arg (HMDB0000517) | 12.6% | 9.7% | 7.9% | 220.4% | 19.7% | 219.5% | 4.45 | 9.80 | 2.84 | 3.65 | 4.91 | 2 | 0.03% | 0.33 | 0.996748222 | Arg |
| Guanidinosuccinate (HMDB0003157) | 6.4% | 3.2% | 5.5% | 48.8% | 13.6% | 47.0% | 6.39 | 3.12 | 4.29 | 5.84 | 7.90 | 3 | 0.04% | 0.34 | 0.982547982 | Guanidinosuccinate |
| Indole-3-acetate (HMDB0000197) | 29.5% | 20.8% | 21.1% | 69.8% | 25.6% | 64.9% | 7.56 | 5.27 | 4.60 | 6.49 | 9.10 | 166 | 2.47% | 0.75 | 0.821115979 | Indole-3-acetate |
| Citrulline (HMDB0000904) | 30.9% | 17.0% | 25.4% | 217.2% | 27.5% | 215.5% | 1.36 | 2.95 | 0.68 | 1.00 | 1.52 | 410 | 6.10% | 0.13 | 0.979728174 | Citrulline |
| Tyr (HMDB0000158) | 6.2% | 3.7% | 5.0% | 87.3% | 11.1% | 86.6% | 14.24 | 12.44 | 9.47 | 12.82 | 17.18 | 1 | 0.01% | 2.41 | 0.99491601 | Tyr |
| SDMA (HMDB0003334) | 9.6% | 6.7% | 7.0% | 21.0% | 11.3% | 18.0% | 7.82 | 1.64 | 6.69 | 7.64 | 8.82 | 0 | 0.00% | 1.47 | 0.792704941 | SDMA |
| ADMA (HMDB0001539) | 9.1% | 6.6% | 6.5% | 23.7% | 10.6% | 21.5% | 7.82 | 1.86 | 6.54 | 7.67 | 8.92 | 0 | 0.00% | 2.65 | 0.854074091 | ADMA |
| o-Acetylcarnitine (HMDB0000201) | 10.6% | 7.3% | 7.8% | 131.9% | 18.5% | 130.6% | 7.67 | 10.12 | 2.13 | 4.82 | 9.28 | 76 | 1.13% | 0.14 | 0.993489589 | o-Acetylcarnitine |
| Trp (HMDB00929) | 6.0% | 3.8% | 4.7% | 50.6% | 9.7% | 49.7% | 11.31 | 5.73 | 7.90 | 10.43 | 13.67 | 1 | 0.01% | 1.37 | 0.985779265 | Trp |
| Cystine (HMDB0000192) | 7.0% | 3.7% | 6.0% | 105.2% | 15.6% | 104.1% | 12.85 | 13.52 | 7.27 | 9.66 | 13.98 | 1 | 0.01% | 1.86 | 0.995517757 | Cystine |
| Uridine (HMDB0000296) | 11.7% | 9.3% | 6.9% | 18.4% | 11.2% | 14.8% | 81.03 | 14.92 | 71.09 | 80.34 | 90.09 | 3 | 0.04% | 9.90 | 0.599297901 | Uridine |
| Adenosine (HMDB0000050) | 19.8% | 9.8% | 17.3% | 37.0% | 12.6% | 34.8% | 1.23 | 0.45 | 0.96 | 1.23 | 1.49 | 218 | 3.24% | 0.08 | 0.714833463 | Adenosine |
| Ethanolamine (HMDB0000149) | 9.6% | 5.1% | 8.2% | 26.1% | 9.9% | 24.3% | 23.82 | 6.22 | 19.60 | 23.45 | 27.67 | 0 | 0.00% | 3.11 | 0.866093238 | Ethanolamine |
| Piperidine (HMDB0034301) | 9.3% | 6.3% | 6.8% | 158.7% | 22.3% | 157.2% | 1.89 | 3.00 | 0.28 | 0.77 | 2.31 | 90 | 1.34% | 0.02 | 0.996581205 | Piperidine |
| 1-Methylhistamine (HMDB0000898) | 29.5% | 15.8% | 25.5% | 47.7% | 18.2% | 43.8% | 0.27 | 0.13 | 0.19 | 0.25 | 0.33 | 296 | 4.40% | 0.06 | 0.61603758 | 1-Methylhistamine |
| Imidazole-4-acetate (HMDB0002024) | 16.9% | 6.9% | 15.4% | 74.2% | 15.4% | 72.5% | 1.10 | 0.82 | 0.79 | 1.07 | 1.39 | 763 | 11.35% | 0.19 | 0.947815044 | Imidazole-4-acetate |
| N-Acetylputrescine (HMDB0002064) | 13.1% | 10.1% | 8.4% | 33.1% | 14.5% | 30.0% | 6.27 | 2.07 | 4.80 | 5.98 | 7.41 | 0 | 0.00% | 1.59 | 0.842812883 | N-Acetylputrescine |
| 5-Aminolevulinate (HMDB0001149) | 16.1% | 7.3% | 14.3% | 30.0% | 11.1% | 28.3% | 0.95 | 0.29 | 0.77 | 0.92 | 1.11 | 19 | 0.28% | 0.19 | 0.712193936 | 5-Aminolevulinate |
| gamma-Guanidinobutyrate (HMDB0003464) | 16.1% | 0.07 | 0.14 | 141.3% | 27.8% | 138.6% | 0.69 | 0.98 | 0.35 | 0.49 | 0.74 | 317 | 4.72% | 0.09 | 0.987074065 | gamma-Guanidinobutyrate |
| 4-(beta-Acetylaminoethyl)imidazole (HMDB0013253) | 33.9% | 0.22 | 0.26 | 203.3% | 18.2% | 202.5% | 0.71 | 1.45 | 0.03 | 0.32 | 0.80 | 2250 | 33.48% | 0.05 | 0.972121799 | 4-(beta-Acetylaminoethyl)imidazole |
| Allantoin (HMDB0000462) | 63.9% | 55.6% | 17.2% | 57.3% | 45.8% | 27.3% | 92.03 | 52.72 | 61.32 | 81.18 | 109.98 | 0 | 0.00% | 4.28 | -0.242548204 | Allantoin |
| 7-Methylguanine (HMDB0000897) | 11.5% | 8.1% | 8.1% | 32.5% | 18.6% | 26.5% | 4.79 | 1.56 | 3.76 | 4.90 | 5.84 | 0 | 0.00% | 0.42 | 0.874432442 | 7-Methylguanine |
| N1-Acetylspermidine (HMDB0001276) | 24.5% | 20.4% | 12.8% | 45.8% | 18.1% | 42.0% | 1.76 | 0.81 | 1.22 | 1.62 | 2.13 | 2 | 0.03% | 0.09 | 0.715064311 | N1-Acetylspermidine |
| N8-Acetylspermidine (HMDB0002189) | 22.4% | 18.8% | 13.1% | 36.0% | 18.7% | 30.5% | 1.20 | 0.43 | 0.90 | 1.14 | 1.43 | 0 | 0.00% | 0.07 | 0.613812816 | N8-Acetylspermidine |
| Gly-Leu (HMDB0000759) | 12.5% | 5.2% | 11.4% | 36.1% | 12.0% | 34.1% | 0.95 | 0.34 | 0.73 | 0.90 | 1.11 | 18 | 0.27% | 0.19 | 0.879513913 | Gly-Leu |
| N-epsilon-Acetyllysine (HMDB0000206) | 10.4% | 4.2% | 9.5% | 28.6% | 11.0% | 26.6% | 2.63 | 0.75 | 2.13 | 2.56 | 3.05 | 4 | 0.06% | 0.66 | 0.869114183 | N-epsilon-Acetyllysine |
| N6,N6,N6-Trimethyllysine (HMDB0001325) | 8.9% | 6.8% | 5.7% | 45.0% | 11.9% | 43.5% | 10.59 | 4.77 | 7.92 | 9.43 | 11.79 | 0 | 0.00% | 2.33 | 0.961226175 | N6,N6,N6-Trimethyllysine |
| N-Acetylhistidine (HMDB0032055) | 17.1% | 6.2% | 16.0% | 37.7% | 11.1% | 36.2% | 1.82 | 0.69 | 1.40 | 1.74 | 2.15 | 3 | 0.04% | 0.34 | 0.795499838 | N-Acetylhistidine |
| SAM+ (HMDB0001185) | 15.5% | 8.8% | 12.8% | 29.8% | 15.5% | 25.5% | 4.47 | 1.33 | 3.56 | 4.39 | 5.25 | 21 | 0.31% | 0.67 | 0.727310986 | SAM+ |
| N-Acetylglucosamine (HMDB0000803) | 16.7% | 11.8% | 11.5% | 30.6% | 11.5% | 28.4% | 17.84 | 5.45 | 14.71 | 17.21 | 20.18 | 3 | 0.04% | 3.49 | 0.700853205 | N-Acetylglucosamine |
| Cystathionine (HMDB0000099) | 61.1% | 42.1% | 44.2% | 554.6% | 39.5% | 553.2% | 2.00 | 11.09 | 0.16 | 0.16 | 3.10 | 3658 | 54.43% | 0.31 | 0.987842937 | Cystathionine |
| 7,8-Dihydrobiopterin (HMDB0000038) | 33.8% | 20.9% | 28.1% | 51.6% | 20.1% | 48.1% | 1.81 | 0.93 | 1.32 | 1.85 | 2.37 | 865 | 12.87% | 0.28 | 0.572106733 | 7,8-Dihydrobiopterin |
| 1-Methyladenosine (HMDB0003331) | 14.8% | 10.2% | 10.8% | 24.9% | 15.2% | 20.2% | 5.31 | 1.32 | 4.37 | 5.18 | 6.11 | 3 | 0.04% | 0.75 | 0.648183176 | 1-Methyladenosine |
| Mean for cations | 16.9% | 10.5% | 12.6% | 124.9% | 18.5% | 122.4% | 19.23 | 16.02 | 10.69 | 15.17 | 23.71 | 285 | | 1.4 | 0.9 | |
| Median for cations | 11.7% | 6.9% | 9.0% | 81.3% | 15.6% | 79.9% | 6.39 | 5.45 | 3.76 | 5.31 | 7.72 | 4 | | 0.4 | 1.0 | |

| Metabolite name (HMDB ID) | QC samples | | | Participant samples | | | | | | | | | | | Estimated ICC* | Metabolite name |
|---|---|---|---|---|---|---|---|---|---|---|---|---|---|---|---|---|
| | Total CV | Inter-batch CV | Intra-batch CV | Total CV | Inter-batch CV | Intra-batch CV | Mean (μmol/L) | SD | 1st quartile | Median | 3rd Quartile | | % of ND | Lowest detection value | | |
| **Anions** | | | | | | | | | | | | | | | | |
| Lactate (HMDB0000190) | 26.6% | 20.1% | 17.1% | 74.3% | 30.1% | 67.5% | 18.13 | 13.48 | 11.33 | 16.43 | 21.96 | 0 | 0.00% | 1.46 | 0.871768889 | Lactate |
| Malonate (HMDB0000691) | 593.3% | 124.0% | 580.8% | 78.8% | 35.2% | 71.0% | 1.16 | 0.91 | 0.68 | 1.07 | 1.49 | 1136 | 16.90% | 0.20 | -55.67208663 | Malonate |
| 3-Hydroxybutyrate (HMDB0000011) | 11.9% | 8.1% | 8.3% | 158.8% | 12.1% | 158.4% | 11.23 | 17.83 | 6.46 | 8.70 | 12.14 | 2 | 0.03% | 1.29 | 0.994387461 | 3-Hydroxybutyrate |
| 4-Oxopentanoate (HMDB0000720) | 23.2% | 16.2% | 16.8% | 39.5% | 19.7% | 34.4% | 3.38 | 1.33 | 2.47 | 3.18 | 4.08 | 39 | 0.58% | 0.41 | 0.655538811 | 4-Oxopentanoate |
| Succinate (HMDB0000254) | 17.7% | 14.0% | 11.2% | 55.9% | 24.9% | 50.1% | 11.20 | 6.26 | 7.02 | 9.89 | 13.80 | 1 | 0.01% | 1.43 | 0.900218081 | Succinate |
| 2-Hydroxypentanoate (HMDB0001863) | 15.0% | 11.2% | 9.8% | 50.2% | 15.7% | 47.6% | 15.09 | 7.57 | 10.54 | 13.80 | 18.20 | 0 | 0.00% | 0.88 | 0.910735034 | 2-Hydroxypentanoate |
| Isethionate (HMDB0003903) | 10.8% | 7.3% | 8.0% | 26.6% | 12.9% | 23.1% | 18.02 | 4.80 | 14.78 | 17.46 | 20.55 | 0 | 0.00% | 6.25 | 0.834180243 | Isethionate |
| 5-Oxoproline (HMDB0000267) | 11.5% | 7.9% | 8.3% | 25.7% | 12.4% | 22.2% | 23.42 | 6.02 | 19.60 | 22.63 | 26.32 | 0 | 0.00% | 8.01 | 0.799552619 | 5-Oxoproline |
| 4-Methyl-2-oxopentanoate (HMDB0000695) | 20.3% | 10.6% | 17.1% | 51.3% | 16.8% | 48.4% | 1.79 | 0.92 | 1.28 | 1.67 | 2.16 | 273 | 4.06% | 0.24 | 0.843341667 | 4-Methyl-2-oxopentanoate |
| Glutarate (HMDB0000661) | 52.0% | 46.4% | 20.9% | 76.2% | 53.4% | 53.0% | 14.35 | 10.94 | 8.07 | 11.10 | 16.19 | 0 | 0.00% | 1.54 | 0.535700618 | Glutarate |
| Malate (HMDB0000156) | 21.2% | 16.7% | 13.2% | 236.3% | 33.4% | 234.2% | 1.25 | 2.96 | 0.57 | 0.90 | 1.42 | 135 | 2.01% | 0.11 | 0.991971977 | Malate |
| Threonate (HMDB0000943) | 13.2% | 10.5% | 8.0% | 26.3% | 14.5% | 21.7% | 72.45 | 19.06 | 60.35 | 70.21 | 82.25 | 0 | 0.00% | 24.72 | 0.749961247 | Threonate |
| Ethanolamine phosphate (HMDB0000224) | 15.4% | 9.4% | 12.2% | 61.6% | 20.8% | 58.0% | 3.89 | 2.39 | 2.40 | 3.58 | 4.99 | 386 | 5.74% | 0.29 | 0.937921696 | Ethanolamine phosphate |
| 2-Oxoglutarate (HMDB0000208) | 69.7% | 14.6% | 68.0% | 79.0% | 24.1% | 75.0% | 26.74 | 21.12 | 13.59 | 21.56 | 34.36 | 213 | 3.17% | 1.95 | 0.222172024 | 2-Oxoglutarate |
| Pimelate (HMDB0000857) | 12.8% | 11.0% | 7.5% | 65.4% | 23.1% | 61.3% | 8.00 | 5.23 | 4.52 | 6.64 | 9.87 | 0 | 0.00% | 1.06 | 0.961487841 | Pimelate |
| Urate (HMDB0000289) | 13.2% | 10.8% | 6.9% | 42.3% | 30.4% | 28.5% | 412.45 | 174.57 | 282.52 | 395.63 | 517.79 | 0 | 0.00% | 54.10 | 0.903362717 | Urate |
| Glycerophosphate (HMDB0000126) | 13.8% | 9.1% | 9.8% | 62.3% | 20.9% | 58.6% | 7.66 | 4.77 | 4.43 | 6.70 | 9.75 | 73 | 1.09% | 0.46 | 0.95092717 | Glycerophosphate |
| trans-Aconitate (HMDB0000958) | 19.2% | 17.1% | 10.7% | 44.0% | 26.1% | 36.2% | 5.72 | 2.52 | 4.38 | 5.22 | 6.40 | 0 | 0.00% | 1.86 | 0.809958881 | trans-Aconitate |
| cis-Aconitate (HMDB0000072) | 15.7% | 10.9% | 11.4% | 31.0% | 16.8% | 25.9% | 32.60 | 10.10 | 25.46 | 31.32 | 38.01 | 0 | 0.00% | 8.51 | 0.744587592 | cis-Aconitate |
| N-Acetylaspartate (HMDB0000812) | 17.1% | 14.5% | 9.9% | 38.9% | 19.6% | 33.6% | 7.71 | 3.00 | 5.42 | 7.30 | 9.55 | 0 | 0.00% | 0.79 | 0.80704322 | N-Acetylaspartate |
| Hippurate (HMDB0000714) | 14.6% | 12.7% | 7.4% | 94.2% | 19.2% | 92.2% | 251.97 | 237.27 | 90.71 | 187.27 | 335.87 | 3 | 0.04% | 3.05 | 0.975938746 | Hippurate |
| Homovanillate (HMDB0000118) | 21.0% | 20.0% | 7.7% | 93.3% | 20.9% | 90.8% | 42.99 | 40.09 | 16.21 | 30.08 | 56.03 | 2 | 0.03% | 2.28 | 0.949318458 | Homovanillate |
| 4-Pyridoxate (HMDB0000017) | 22.2% | 18.1% | 13.0% | 130.1% | 24.2% | 127.9% | 2.05 | 2.67 | 0.96 | 1.37 | 2.11 | 1 | 0.01% | 0.13 | 0.970999032 | 4-Pyridoxate |
| Azelate (HMDB0000784) | 18.0% | 10.5% | 14.5% | 76.1% | 27.2% | 71.0% | 1.18 | 0.90 | 0.71 | 0.98 | 1.36 | 20 | 0.30% | 0.14 | 0.943991116 | Azelate |
| Isocitrate (HMDB0000193) | 11.8% | 9.6% | 7.1% | 24.9% | 13.5% | 21.0% | 53.11 | 13.25 | 43.78 | 52.15 | 61.11 | 0 | 0.00% | 13.45 | 0.77549542 | Isocitrate |
| Citrate (HMDB0000094) | 16.3% | 15.2% | 5.6% | 54.9% | 24.1% | 49.1% | 439.20 | 241.19 | 264.61 | 399.42 | 574.42 | 0 | 0.00% | 1.05 | 0.9116103 | Citrate |
| Quinate (HMDB0003072) | 12.2% | 9.1% | 8.7% | 112.5% | 20.9% | 110.6% | 13.68 | 15.39 | 3.02 | 9.03 | 19.27 | 367 | 5.46% | 0.14 | 0.988163591 | Quinate |
| Glucuronate (HMDB0000127) | 16.1% | 10.8% | 11.3% | 34.5% | 16.4% | 30.3% | 32.02 | 11.06 | 24.91 | 30.05 | 36.92 | 18 | 0.27% | 1.35 | 0.782957119 | Glucuronate |
| Cysteine S-sulfate (HMDB0000731) | 47.5% | 27.3% | 39.0% | 52.3% | 22.3% | 47.2% | 1.66 | 0.87 | 1.17 | 1.55 | 2.03 | 214 | 3.18% | 0.21 | 0.175021325 | Cysteine S-sulfate |
| Mucate (HMDB0000639) | 19.4% | 12.7% | 14.6% | 68.6% | 27.4% | 63.3% | 2.40 | 1.65 | 1.56 | 2.07 | 2.74 | 44 | 0.65% | 0.37 | 0.920358256 | Mucate |
| 3-Indoxyl sulfate (HMDB0000682) | 13.1% | 11.1% | 6.9% | 66.9% | 19.7% | 64.0% | 56.45 | 37.79 | 30.81 | 48.20 | 71.85 | 1 | 0.01% | 1.15 | 0.961903435 | 3-Indoxyl sulfate |
| Glycolate (HMDB0000115) | 21.3% | 14.6% | 15.6% | 45.1% | 19.3% | 40.8% | 20.45 | 9.22 | 14.86 | 19.53 | 24.94 | 231 | 3.44% | 2.16 | 0.776354023 | Glycolate |
| Oxamate (No HMDB ID, CAS number 471-47-6) | 21.2% | 13.4% | 16.9% | 104.4% | 23.5% | 101.8% | 3.78 | 3.94 | 1.81 | 3.12 | 4.81 | 1106 | 16.46% | 0.53 | 0.958810253 | Oxamate |
| 3-Hydroxypropionate (HMDB0000700) | 361.2% | 77.6% | 352.6% | 102.3% | 36.2% | 96.0% | 2.86 | 2.92 | 0.34 | 2.53 | 4.30 | 2530 | 37.65% | 0.68 | -11.45715295 | 3-Hydroxypropionate |
| 2-Hydroxyisobutyrate (HMDB0000729) | 14.3% | 9.7% | 10.4% | 33.6% | 12.2% | 31.1% | 4.88 | 1.64 | 3.74 | 4.69 | 5.82 | 2 | 0.03% | 0.36 | 0.81907729 | 2-Hydroxyisobutyrate |
| Itaconate (HMDB0002092) | 792.9% | 139.5% | 780.8% | 74.3% | 25.0% | 70.3% | 0.32 | 0.24 | 0.18 | 0.30 | 0.44 | 1334 | 19.85% | 0.05 | -112.888247 | Itaconate |
| N-Acetyl-beta-alanine (HMDB0061880) | 16.0% | 10.5% | 11.7% | 62.2% | 16.2% | 60.0% | 2.10 | 1.31 | 1.26 | 1.80 | 2.59 | 58 | 0.86% | 0.27 | 0.933830676 | N-Acetyl-beta-alanine |
| 3-Ureidopropionate (HMDB0000026) | 69.1% | 24.3% | 64.8% | 124.5% | 14.4% | 123.7% | 2.64 | 3.29 | 1.82 | 2.43 | 3.18 | 549 | 8.17% | 0.41 | 0.692130085 | 3-Ureidopropionate |
| Adipate (HMDB0000448) | 14.8% | 9.7% | 11.3% | 55.4% | 19.3% | 52.1% | 4.42 | 2.45 | 2.87 | 3.86 | 5.31 | 32 | 0.48% | 0.83 | 0.929059804 | Adipate |
| p-Hydroxyphenylacetate (HMDB0000020) | 20.5% | 17.2% | 12.0% | 94.4% | 23.2% | 91.5% | 22.90 | 21.62 | 11.89 | 17.66 | 27.02 | 185 | 2.75% | 0.98 | 0.952769635 | p-Hydroxyphenylacetate |
| 3-Hydroxy-3-methylglutarate (HMDB0000355) | 21.9% | 15.3% | 16.1% | 35.4% | 20.6% | 29.0% | 3.32 | 1.18 | 2.49 | 3.17 | 3.94 | 0 | 0.00% | 0.83 | 0.618311997 | 3-Hydroxy-3-methylglutarate |
| 2,3-Pyridinedicarboxylate (HMDB0000232) | 19.0% | 13.0% | 13.8% | 38.9% | 16.7% | 35.2% | 4.97 | 1.94 | 3.70 | 4.66 | 5.90 | 36 | 0.54% | 1.10 | 0.762349564 | 2,3-Pyridinedicarboxylate |
| 4-Hydroxymandelate (HMDB0000822) | 26.8% | 18.9% | 18.9% | 42.3% | 19.2% | 37.7% | 3.22 | 1.36 | 2.37 | 2.99 | 3.79 | 65 | 0.97% | 0.91 | 0.599217681 | 4-Hydroxymandelate |
| Suberate (HMDB0000893) | 16.3% | 13.1% | 10.6% | 69.1% | 19.8% | 66.2% | 2.93 | 2.02 | 1.66 | 2.42 | 3.56 | 5 | 0.07% | 0.38 | 0.944104859 | Suberate |
| Allantoate (HMDB0001209) | 33.4% | 13.0% | 30.7% | 118.9% | 27.0% | 115.9% | 2.66 | 3.16 | 0.42 | 1.93 | 3.52 | 1703 | 25.34% | 0.20 | 0.921276701 | Allantoate |
| 3PG (HMDB0000807) | 483.4% | 105.4% | 471.9% | 91.3% | 40.8% | 81.8% | 0.78 | 0.71 | 0.08 | 0.76 | 1.21 | 2647 | 39.39% | 0.17 | -27.03647667 | 3PG |
| N-Acetylglutamate (HMDB0001138) | 14.3% | 9.8% | 10.6% | 36.5% | 14.6% | 33.5% | 2.95 | 1.07 | 2.19 | 2.78 | 3.50 | 1 | 0.01% | 0.76 | 0.845217861 | N-Acetylglutamate |
| o-Hydroxyhippurate (HMDB0000840) | 643.2% | 71.6% | 639.3% | 671.2% | 16.1% | 671.0% | 2.56 | 17.19 | 0.36 | 0.66 | 1.20 | 1097 | 16.32% | 0.10 | 0.081584033 | o-Hydroxyhippurate |
| Gluconate (HMDB0000625) | 14.4% | 11.9% | 9.4% | 35.8% | 19.0% | 30.8% | 63.45 | 22.73 | 47.69 | 60.98 | 76.40 | 41 | 0.61% | 7.70 | 0.837651271 | Gluconate |
| 4-Hydroxy-3-methoxymandelate (HMDB0000291) | 19.6% | 15.5% | 12.7% | 35.4% | 17.1% | 31.2% | 4.46 | 1.58 | 3.37 | 4.25 | 5.33 | 11 | 0.16% | 0.45 | 0.693762176 | 4-Hydroxy-3-methoxymandelate |
| Saccharate (HMDB0000663) | 16.5% | 11.5% | 11.6% | 48.9% | 14.3% | 46.9% | 5.11 | 2.50 | 3.94 | 4.76 | 5.74 | 2 | 0.03% | 1.25 | 0.88662064 | Saccharate |
| Pantothenate (HMDB0000210) | 14.0% | 9.5% | 10.5% | 100.1% | 19.0% | 98.3% | 3.02 | 3.02 | 1.76 | 2.55 | 3.56 | 50 | 0.74% | 0.21 | 0.980316365 | Pantothenate |
| Biotin (HMDB0000030) | 44.4% | 30.3% | 33.2% | 226.6% | 151.6% | 174.0% | 1.08 | 2.45 | 0.07 | 0.07 | 1.30 | 3634 | 54.08% | 0.13 | 0.961656171 | Biotin |
| N-Acetylneuraminate (HMDB0000230) | 17.6% | 18.4% | 7.2% | 21.7% | 9.1% | 19.6% | 18.15 | 3.93 | 15.58 | 17.94 | 20.44 | 14 | 0.21% | 1.67 | 0.338893039 | N-Acetylneuraminate |
| Mean for anions | 72.6% | 22.6% | 66.0% | 81.5% | 24.0% | 76.2% | 32.9 | 19.1 | 20.2 | 29.3 | 41.3 | 338 | | 3.0 | -3.1 | |
| Median for anions | 17.8% | 13.1% | 11.7% | 61.9% | 19.8% | 55.5% | 5.0 | 3.2 | 3.7 | 4.7 | 5.9 | 19 | | 0.8 | 0.8 | |

CV: coefficient of variation, ICC: intraclass correlation coefficient, ND: not detectable values, QC: quality control, SD: standard deviation

*ICC was estimated by following; 1 - (Total CV of QC samples )^2 / (Total CV of subject samples )^2

We indentified urinary metabolites which standard reagents were available, so all metabolites were classified in level 1 for guidelines by the Metabolomcis Standards Initiative.

Salek, R. M., Steinbeck, C., Viant, M. R., Goodacre, R., & Dunn, W. B. (2013). The role of reporting standards for metabolite annotation and identification in metabolomic studies. GigaScience, 2(1), 13. https://doi.org/10.1186/2047-217X-2-13

**Table S3. Statistical summary of measured metabolites by exclusion with the creatinine revels. (excluding samples of creatinine >3.0 g / L or <0.3 g / L in participants)**

| Metabolite name | Participant samples | | | | | | | | | | | Estimated ICC* | Metabolite name |
|---|---|---|---|---|---|---|---|---|---|---|---|---|---|
| | Total CV | Inter-batch CV | Intra-batch CV | Mean (µmol/L) | SD | 1st quartile | Median | 3rd Quartile | Number of ND | % of ND | Lowest detection value | | |
| **Cations** | | | | | | | | | | | | | Cations |
| Gly | 68.5% | 20.3% | 65.6% | 76.03 | 52.11 | 42.52 | 61.59 | 91.97 | 0 | 0.00% | 5.11 | 0.993349572 | Gly |
| Trimethylamine N-oxide | 114.3% | 29.4% | 110.6% | 64.71 | 73.96 | 21.89 | 36.96 | 78.23 | 19 | 0.29% | 0.11 | 0.991258302 | Trimethylamine N-oxide |
| beta-Ala | 91.1% | 14.8% | 89.9% | 1.27 | 1.16 | 0.67 | 1.15 | 1.70 | 1054 | 15.98% | 0.19 | 0.961319517 | beta-Ala |
| Ala | 69.5% | 13.6% | 68.2% | 21.23 | 14.76 | 13.24 | 18.10 | 25.53 | 0 | 0.00% | 3.65 | 0.99237698 | Ala |
| 3-Aminoisobutyrate | 114.4% | 12.0% | 113.8% | 49.80 | 57.00 | 6.23 | 18.89 | 95.15 | 0 | 0.00% | 0.43 | 0.996784154 | 3-Aminoisobutyrate |
| 2AB | 82.9% | 13.3% | 81.8% | 1.10 | 0.92 | 0.76 | 1.02 | 1.36 | 291 | 4.41% | 0.22 | 0.945164405 | 2AB |
| N,N-Dimethylglycine | 326.7% | 15.7% | 326.3% | 6.97 | 22.78 | 3.68 | 5.35 | 7.76 | 13 | 0.20% | 0.57 | 0.999191481 | N,N-Dimethylglycine |
| Choline | 49.6% | 14.6% | 47.4% | 3.52 | 1.75 | 2.48 | 3.22 | 4.12 | 0 | 0.00% | 0.85 | 0.933909543 | Choline |
| Ser | 54.6% | 11.2% | 53.5% | 33.11 | 18.07 | 23.28 | 29.65 | 38.88 | 34 | 0.52% | 0.40 | 0.988525207 | Ser |
| Pro | 142.6% | 19.9% | 141.3% | 0.94 | 1.34 | 0.56 | 0.76 | 1.03 | 486 | 7.37% | 0.15 | 0.983918362 | Pro |
| Guanidinoacetate | 49.5% | 15.3% | 47.2% | 59.71 | 29.56 | 37.89 | 54.78 | 76.45 | 0 | 0.00% | 2.41 | 0.983107532 | Guanidinoacetate |
| Val | 287.3% | 13.4% | 287.0% | 4.29 | 12.33 | 3.28 | 4.01 | 4.84 | 109 | 1.65% | 0.26 | 0.997098734 | Val |
| Betaine | 148.1% | 21.4% | 146.7% | 20.15 | 29.85 | 8.84 | 12.87 | 20.39 | 21 | 0.32% | 2.11 | 0.994339087 | Betaine |
| Thr | 95.1% | 16.9% | 93.6% | 15.72 | 14.95 | 9.35 | 12.78 | 18.16 | 2 | 0.03% | 1.31 | 0.997119994 | Thr |
| Taurine | 69.7% | 15.1% | 68.0% | 173.52 | 120.94 | 97.75 | 151.32 | 218.03 | 5 | 0.08% | 6.33 | 0.969845027 | Taurine |
| Creatine | 152.6% | 40.0% | 147.3% | 73.99 | 112.91 | 7.79 | 21.39 | 95.43 | 0 | 0.00% | 1.70 | 0.998703093 | Creatine |
| Ile | 1386.6% | 63.8% | 1385.2% | 1.53 | 21.21 | 0.95 | 1.18 | 1.47 | 47 | 0.71% | 0.06 | 0.999572885 | Ile |
| Leu | 366.8% | 12.5% | 366.6% | 3.51 | 12.89 | 2.56 | 3.16 | 3.89 | 37 | 0.56% | 0.10 | 0.999078927 | Leu |
| Asn | 81.0% | 14.2% | 79.8% | 12.02 | 9.74 | 7.94 | 10.32 | 13.87 | 14 | 0.21% | 1.79 | 0.987410703 | Asn |
| Ornithine | 178.7% | 18.5% | 177.7% | 1.95 | 3.49 | 1.17 | 1.53 | 2.05 | 40 | 0.61% | 0.33 | 0.993342958 | Ornithine |
| Asp | 338.0% | 51.2% | 334.1% | 0.30 | 1.03 | 0.10 | 0.10 | 0.10 | 5447 | 82.59% | 0.19 | 0.840943913 | Asp |
| Hypoxanthine | 49.8% | 17.5% | 46.7% | 13.10 | 6.52 | 8.90 | 12.32 | 16.24 | 2 | 0.03% | 1.03 | 0.980228623 | Hypoxanthine |
| 1-Methylnicotinamide | 46.4% | 16.6% | 43.2% | 10.45 | 4.85 | 7.03 | 9.76 | 13.04 | 0 | 0.00% | 0.65 | 0.880674721 | 1-Methylnicotinamide |
| Trigonelline | 93.3% | 20.1% | 91.1% | 16.17 | 15.08 | 6.59 | 11.37 | 20.51 | 1 | 0.02% | 0.51 | 0.985265861 | Trigonelline |
| Urocanate | 56.7% | 20.5% | 53.0% | 2.29 | 1.30 | 1.64 | 2.26 | 2.96 | 781 | 11.84% | 0.65 | 0.781228258 | Urocanate |
| Proline betaine | 145.8% | 32.1% | 142.4% | 15.35 | 22.39 | 3.67 | 6.88 | 17.10 | 4 | 0.06% | 0.62 | 0.993768817 | Proline betaine |
| gamma-Butyrobetaine | 112.6% | 17.7% | 111.3% | 0.36 | 0.40 | 0.09 | 0.27 | 0.49 | 1556 | 23.59% | 0.04 | 0.962976242 | gamma-Butyrobetaine |
| Gln | 84.2% | 14.2% | 83.0% | 61.44 | 51.72 | 42.29 | 55.65 | 73.36 | 2 | 0.03% | 5.67 | 0.995064681 | Gln |
| Lys | 140.7% | 17.0% | 139.7% | 41.27 | 58.06 | 15.45 | 26.45 | 47.53 | 0 | 0.00% | 2.52 | 0.998381984 | Lys |
| Glu | 65.5% | 12.9% | 64.2% | 2.32 | 1.52 | 1.62 | 2.17 | 2.79 | 165 | 2.50% | 0.37 | 0.941677214 | Glu |
| Met | 179.4% | 12.5% | 178.9% | 1.46 | 2.63 | 1.08 | 1.37 | 1.72 | 230 | 3.49% | 0.28 | 0.976140069 | Met |
| His | 55.0% | 9.2% | 54.2% | 86.62 | 47.63 | 54.76 | 77.81 | 108.53 | 0 | 0.00% | 2.56 | 0.98602169 | His |
| alpha-Aminoadipate | 119.8% | 13.2% | 118.9% | 4.65 | 5.57 | 3.23 | 4.26 | 5.53 | 10 | 0.15% | 0.40 | 0.995204782 | alpha-Aminoadipate |
| Carnitine | 94.2% | 17.2% | 92.7% | 18.45 | 17.38 | 5.67 | 13.33 | 25.97 | 0 | 0.00% | 0.32 | 0.983605414 | Carnitine |
| Phe | 96.4% | 9.7% | 96.0% | 6.93 | 6.69 | 5.12 | 6.40 | 8.12 | 0 | 0.00% | 0.63 | 0.994801941 | Phe |
| 3-Methylhistidine | 88.5% | 11.9% | 87.7% | 81.51 | 72.12 | 38.50 | 55.96 | 97.59 | 0 | 0.00% | 12.69 | 0.996279879 | 3-Methylhistidine |
| Arg | 220.8% | 19.1% | 220.1% | 4.48 | 9.89 | 2.86 | 3.68 | 4.92 | 2 | 0.03% | 0.33 | 0.996697467 | Arg |
| Guanidinosuccinate | 48.7% | 13.3% | 47.0% | 6.42 | 3.12 | 4.32 | 5.87 | 7.93 | 3 | 0.05% | 0.64 | 0.981411053 | Guanidinosuccinate |
| Indole-3-acetate | 68.8% | 24.5% | 64.3% | 7.64 | 5.26 | 4.69 | 6.55 | 9.15 | 164 | 2.49% | 1.38 | 0.81480508 | Indole-3-acetate |
| Citrulline | 216.8% | 26.6% | 215.2% | 1.37 | 2.97 | 0.69 | 1.01 | 1.54 | 409 | 6.20% | 0.13 | 0.98075389 | Citrulline |
| Tyr | 87.3% | 10.4% | 86.7% | 14.34 | 12.52 | 9.56 | 12.93 | 17.28 | 1 | 0.02% | 2.41 | 0.994894278 | Tyr |
| SDMA | 20.9% | 11.3% | 17.9% | 7.85 | 1.64 | 6.71 | 7.66 | 8.84 | 0 | 0.00% | 1.47 | 0.796795872 | SDMA |
| ADMA | 23.5% | 10.3% | 21.4% | 7.86 | 1.85 | 6.59 | 7.71 | 8.96 | 0 | 0.00% | 2.94 | 0.856261687 | ADMA |
| o-Acetylcarnitine | 131.6% | 18.4% | 130.4% | 7.71 | 10.15 | 2.17 | 4.86 | 9.30 | 76 | 1.15% | 0.16 | 0.993364322 | o-Acetylcarnitine |
| Trp | 50.4% | 9.0% | 49.6% | 11.38 | 5.74 | 7.98 | 10.48 | 13.74 | 1 | 0.02% | 1.37 | 0.985437046 | Trp |
| Cystine | 105.1% | 14.7% | 104.1% | 12.96 | 13.62 | 7.34 | 9.74 | 14.11 | 1 | 0.02% | 1.86 | 0.9953189 | Cystine |
| Uridine | 18.3% | 11.0% | 14.8% | 81.32 | 14.84 | 71.45 | 80.59 | 90.29 | 3 | 0.05% | 9.90 | 0.543468372 | Uridine |
| Adenosine | 36.8% | 12.2% | 34.8% | 1.23 | 0.45 | 0.97 | 1.23 | 1.50 | 218 | 3.31% | 0.08 | 0.716747389 | Adenosine |
| Ethanolamine | 25.8% | 9.4% | 24.1% | 23.95 | 6.18 | 19.74 | 23.56 | 27.75 | 0 | 0.00% | 3.11 | 0.865189575 | Ethanolamine |
| Piperidine | 158.1% | 21.4% | 156.7% | 1.91 | 3.02 | 0.29 | 0.79 | 2.33 | 90 | 1.36% | 0.02 | 0.996569486 | Piperidine |
| 1-Methylhistamine | 47.5% | 17.8% | 43.8% | 0.27 | 0.13 | 0.19 | 0.26 | 0.33 | 296 | 4.49% | 0.06 | 0.605177488 | 1-Methylhistamine |
| Imidazole-4-acetate | 74.1% | 15.4% | 72.5% | 1.11 | 0.82 | 0.79 | 1.08 | 1.40 | 762 | 11.55% | 0.19 | 0.94946963 | Imidazole-4-acetate |
| N-Acetylputrescine | 32.9% | 14.3% | 30.0% | 6.30 | 2.08 | 4.83 | 6.01 | 7.44 | 0 | 0.00% | 1.59 | 0.839108254 | N-Acetylputrescine |
| 5-Aminolevulinate | 29.8% | 10.6% | 28.2% | 0.96 | 0.29 | 0.78 | 0.93 | 1.11 | 19 | 0.29% | 0.19 | 0.704123081 | 5-Aminolevulinate |
| gamma-Guanidinobutyrate | 141.3% | 27.4% | 138.7% | 0.70 | 0.99 | 0.35 | 0.50 | 0.75 | 317 | 4.81% | 0.09 | 0.987346841 | gamma-Guanidinobutyrate |
| 4-(beta-Acetylaminoethyl)imidazole | 203.3% | 17.4% | 202.6% | 0.72 | 1.46 | 0.03 | 0.33 | 0.80 | 2213 | 33.56% | 0.05 | 0.972330653 | 4-(beta-Acetylaminoethyl)imidazole |
| Allantoin | 56.8% | 45.3% | 26.4% | 92.92 | 52.79 | 62.48 | 82.01 | 111.32 | 0 | 0.00% | 4.28 | -0.220063994 | Allantoin |
| 7-Methylguanine | 31.7% | 17.6% | 26.1% | 4.84 | 1.53 | 3.82 | 4.94 | 5.86 | 0 | 0.00% | 0.66 | 0.869722504 | 7-Methylguanine |
| N1-Acetylspermidine | 45.8% | 18.1% | 42.0% | 1.77 | 0.81 | 1.22 | 1.62 | 2.13 | 2 | 0.03% | 0.09 | 0.723805471 | N1-Acetylspermidine |
| N8-Acetylspermidine | 36.1% | 18.7% | 30.5% | 1.21 | 0.44 | 0.91 | 1.14 | 1.43 | 0 | 0.00% | 0.07 | 0.618096413 | N8-Acetylspermidine |
| Gly-Leu | 35.8% | 11.5% | 33.9% | 0.96 | 0.34 | 0.73 | 0.91 | 1.12 | 18 | 0.27% | 0.19 | 0.874317102 | Gly-Leu |
| N-epsilon-Acetyllysine | 28.2% | 10.3% | 26.4% | 2.64 | 0.75 | 2.15 | 2.57 | 3.06 | 4 | 0.06% | 0.66 | 0.86348791 | N-epsilon-Acetyllysine |
| N6,N6,N6-Trimethyllysine | 45.0% | 11.6% | 43.5% | 10.64 | 4.78 | 7.96 | 9.47 | 11.84 | 0 | 0.00% | 2.33 | 0.962607806 | N6,N6,N6-Trimethyllysine |
| N-Acetylhistidine | 37.5% | 10.5% | 36.1% | 1.84 | 0.69 | 1.42 | 1.75 | 2.16 | 3 | 0.05% | 0.34 | 0.801819901 | N-Acetylhistidine |
| SAM+ | 29.3% | 14.9% | 25.3% | 4.50 | 1.32 | 3.60 | 4.42 | 5.27 | 21 | 0.32% | 0.67 | 0.720868002 | SAM+ |
| N-Acetylglucosamine | 30.4% | 11.1% | 28.3% | 17.93 | 5.45 | 14.79 | 17.30 | 20.23 | 3 | 0.05% | 3.49 | 0.68731898 | N-Acetylglucosamine |
| Cystathionine | 496.5% | 42.2% | 494.7% | 1.95 | 9.67 | 0.16 | 0.16 | 3.12 | 3583 | 54.33% | 0.31 | 0.98394778 | Cystathionine |
| 7,8-Dihydrobiopterin | 51.4% | 19.8% | 48.0% | 1.82 | 0.93 | 1.34 | 1.87 | 2.38 | 860 | 13.04% | 0.28 | 0.589167112 | 7,8-Dihydrobiopterin |
| 1-Methyladenosine | 24.8% | 15.1% | 20.2% | 5.32 | 1.32 | 4.38 | 5.20 | 6.12 | 3 | 0.05% | 0.75 | 0.648390483 | 1-Methyladenosine |
| Mean for cations | 121.6% | 18.1% | 119.1% | 19.37 | 16.01 | 10.81 | 15.30 | 23.88 | #REF! | | 1.4 | 0.9 | |
| Median for cations | 74.1% | 15.1% | 72.5% | 6.42 | 5.45 | 3.82 | 5.35 | 7.76 | #REF! | | 0.6 | 1.0 | |

| Metabolite name | Participant samples | | | | | | | | | | | Estimated ICC* | Metabolite name |
|---|---|---|---|---|---|---|---|---|---|---|---|---|---|
| | Total CV | Inter-batch CV | Intra-batch CV | Mean (µmol/L) | SD | 1st quartile | Median | 3rd Quartile | | % of ND | Lowest detection value | | |
| **Anions** | | | | | | | | | | | | | |
| Lactate | 74.1% | 30.0% | 67.2% | 18.29 | 13.54 | 11.52 | 16.57 | 22.08 | 0 | 0.00% | 1.46 | 0.870770691 | Lactate |
| Malonate | 78.4% | 35.0% | 70.7% | 1.16 | 0.91 | 0.70 | 1.07 | 1.50 | 1084 | 16.44% | 0.26 | -56.22372501 | Malonate |
| 3-Hydroxybutyrate | 146.3% | 14.0% | 145.7% | 11.19 | 16.37 | 6.51 | 8.74 | 12.21 | 2 | 0.03% | 1.49 | 0.99338983 | 3-Hydroxybutyrate |
| 4-Oxopentanoate | 39.4% | 19.7% | 34.3% | 3.39 | 1.33 | 2.48 | 3.19 | 4.09 | 39 | 0.59% | 0.41 | 0.654138719 | 4-Oxopentanoate |
| Succinate | 55.5% | 24.4% | 49.9% | 11.30 | 6.27 | 7.12 | 9.98 | 13.91 | 1 | 0.02% | 1.43 | 0.898830546 | Succinate |
| 2-Hydroxypentanoate | 49.9% | 15.3% | 47.4% | 15.18 | 7.57 | 10.64 | 13.87 | 18.26 | 0 | 0.00% | 0.88 | 0.909754489 | 2-Hydroxypentanoate |
| Isethionate | 26.6% | 12.8% | 23.1% | 18.08 | 4.80 | 14.87 | 17.52 | 20.62 | 0 | 0.00% | 6.25 | 0.833206743 | Isethionate |
| 5-Oxoproline | 25.5% | 12.2% | 22.0% | 23.50 | 5.99 | 19.73 | 22.71 | 26.37 | 0 | 0.00% | 8.01 | 0.796236713 | 5-Oxoproline |
| 4-Methyl-2-oxopentanoate | 51.0% | 16.7% | 48.1% | 1.80 | 0.92 | 1.29 | 1.67 | 2.17 | 262 | 3.97% | 0.24 | 0.841577373 | 4-Methyl-2-oxopentanoate |
| Glutarate | 75.9% | 53.1% | 52.6% | 14.44 | 10.97 | 8.14 | 11.18 | 16.30 | 0 | 0.00% | 1.54 | 0.532059859 | Glutarate |
| Malate | 236.0% | 32.5% | 234.1% | 1.27 | 2.99 | 0.58 | 0.91 | 1.43 | 126 | 1.91% | 0.12 | 0.99195447 | Malate |
| Threonate | 26.2% | 14.4% | 21.7% | 72.73 | 19.07 | 60.59 | 70.49 | 82.45 | 0 | 0.00% | 24.72 | 0.748153393 | Threonate |
| Ethanolamine phosphate | 61.0% | 20.1% | 57.7% | 3.92 | 2.39 | 2.44 | 3.61 | 5.02 | 354 | 5.37% | 0.49 | 0.936762374 | Ethanolamine phosphate |
| 2-Oxoglutarate | 78.7% | 23.6% | 74.8% | 26.95 | 21.21 | 13.72 | 21.79 | 34.70 | 212 | 3.21% | 1.95 | 0.216415957 | 2-Oxoglutarate |
| Pimelate | 65.2% | 22.9% | 61.1% | 8.04 | 5.24 | 4.57 | 6.66 | 9.91 | 0 | 0.00% | 1.06 | 0.961159849 | Pimelate |
| Urate | 41.2% | 29.2% | 27.9% | 417.59 | 172.04 | 289.25 | 400.12 | 520.87 | 0 | 0.00% | 55.44 | 0.898006428 | Urate |
| Glycerophosphate | 61.7% | 20.1% | 58.3% | 7.73 | 4.77 | 4.52 | 6.77 | 9.84 | 72 | 1.09% | 0.65 | 0.949957421 | Glycerophosphate |
| trans-Aconitate | 44.3% | 26.1% | 36.4% | 5.73 | 2.53 | 4.38 | 5.22 | 6.42 | 0 | 0.00% | 1.86 | 0.812026051 | trans-Aconitate |
| cis-Aconitate | 30.7% | 16.4% | 25.7% | 32.77 | 10.07 | 25.64 | 31.49 | 38.14 | 0 | 0.00% | 8.51 | 0.740133003 | cis-Aconitate |
| N-Acetylaspartate | 38.6% | 19.3% | 33.5% | 7.76 | 2.99 | 5.49 | 7.37 | 9.59 | 0 | 0.00% | 0.79 | 0.804206382 | N-Acetylaspartate |
| Hippurate | 93.9% | 19.2% | 92.0% | 252.78 | 237.45 | 91.05 | 187.74 | 337.45 | 3 | 0.05% | 3.05 | 0.975820071 | Hippurate |
| Homovanillate | 93.3% | 21.1% | 90.8% | 43.13 | 40.22 | 16.27 | 30.17 | 56.20 | 1 | 0.02% | 2.28 | 0.949312576 | Homovanillate |
| 4-Pyridoxate | 130.1% | 23.9% | 127.9% | 2.06 | 2.69 | 0.97 | 1.38 | 2.12 | 1 | 0.02% | 0.13 | 0.970979994 | 4-Pyridoxate |
| Azelate | 75.8% | 26.7% | 70.8% | 1.19 | 0.90 | 0.72 | 0.99 | 1.37 | 20 | 0.30% | 0.14 | 0.943529214 | Azelate |
| Isocitrate | 24.8% | 13.4% | 21.0% | 53.31 | 13.24 | 44.07 | 52.35 | 61.30 | 0 | 0.00% | 13.45 | 0.773578214 | Isocitrate |
| Citrate | 54.3% | 23.6% | 48.8% | 443.31 | 240.93 | 267.57 | 403.30 | 577.46 | 0 | 0.00% | 1.05 | 0.909755198 | Citrate |
| Quinate | 112.5% | 20.5% | 110.7% | 13.67 | 15.38 | 3.02 | 9.03 | 19.26 | 362 | 5.49% | 0.14 | 0.988165196 | Quinate |
| Glucuronate | 34.5% | 16.3% | 30.3% | 32.11 | 11.09 | 24.96 | 30.13 | 37.02 | 18 | 0.27% | 1.35 | 0.783078263 | Glucuronate |
| Cysteine S-sulfate | 51.8% | 21.7% | 46.9% | 1.68 | 0.87 | 1.19 | 1.56 | 2.04 | 206 | 3.12% | 0.21 | 0.158099413 | Cysteine S-sulfate |
| Mucate | 68.6% | 27.1% | 63.4% | 2.42 | 1.66 | 1.57 | 2.09 | 2.76 | 44 | 0.67% | 0.37 | 0.920203762 | Mucate |
| 3-Indoxyl sulfate | 66.7% | 19.4% | 63.9% | 56.82 | 37.92 | 31.19 | 48.52 | 72.45 | 1 | 0.02% | 1.15 | 0.961674795 | 3-Indoxyl sulfate |
| Glycolate | 44.6% | 18.6% | 40.5% | 20.60 | 9.18 | 15.07 | 19.66 | 25.04 | 229 | 3.47% | 2.16 | 0.770910548 | Glycolate |
| Oxamate | 104.3% | 23.1% | 101.9% | 3.81 | 3.97 | 1.83 | 3.15 | 4.84 | 1082 | 16.41% | 0.53 | 0.958765341 | Oxamate |
| 3-Hydroxypropionate | 100.5% | 36.3% | 93.9% | 2.87 | 2.88 | 0.34 | 2.58 | 4.34 | 2461 | 37.32% | 0.68 | -11.92121057 | 3-Hydroxypropionate |
| 2-Hydroxyisobutyrate | 33.4% | 12.1% | 31.0% | 4.90 | 1.64 | 3.75 | 4.71 | 5.83 | 2 | 0.03% | 0.36 | 0.817217735 | 2-Hydroxyisobutyrate |
| Itaconate | 74.3% | 25.0% | 70.4% | 0.32 | 0.24 | 0.18 | 0.30 | 0.44 | 1299 | 19.70% | 0.06 | -112.7457078 | Itaconate |
| N-Acetyl-beta-alanine | 61.8% | 15.8% | 59.7% | 2.12 | 1.31 | 1.28 | 1.82 | 2.61 | 55 | 0.83% | 0.27 | 0.932993505 | N-Acetyl-beta-alanine |
| 3-Ureidopropionate | 124.9% | 14.1% | 124.2% | 2.66 | 3.32 | 1.83 | 2.45 | 3.19 | 525 | 7.96% | 0.41 | 0.694139522 | 3-Ureidopropionate |
| Adipate | 54.9% | 19.3% | 51.5% | 4.44 | 2.44 | 2.89 | 3.89 | 5.32 | 32 | 0.49% | 0.83 | 0.927597833 | Adipate |
| p-Hydroxyphenylacetate | 94.0% | 22.7% | 91.3% | 23.09 | 21.72 | 12.06 | 17.80 | 27.14 | 155 | 2.35% | 0.98 | 0.952398595 | p-Hydroxyphenylacetate |
| 3-Hydroxy-3-methylglutarate | 35.3% | 20.5% | 28.9% | 3.33 | 1.18 | 2.50 | 3.19 | 3.95 | 0 | 0.00% | 0.83 | 0.615547377 | 3-Hydroxy-3-methylglutarate |
| 2,3-Pyridinedicarboxylate | 38.8% | 16.6% | 35.1% | 4.99 | 1.94 | 3.72 | 4.68 | 5.91 | 36 | 0.55% | 1.10 | 0.760516228 | 2,3-Pyridinedicarboxylate |
| 4-Hydroxymandelate | 42.3% | 19.2% | 37.7% | 3.24 | 1.37 | 2.38 | 3.01 | 3.82 | 65 | 0.99% | 0.91 | 0.599127317 | 4-Hydroxymandelate |
| Suberate | 68.8% | 19.6% | 66.1% | 2.94 | 2.03 | 1.67 | 2.43 | 3.57 | 5 | 0.08% | 0.38 | 0.943726521 | Suberate |
| Allantoate | 118.7% | 27.0% | 115.7% | 2.67 | 3.18 | 0.10 | 1.95 | 3.55 | 1653 | 25.06% | 0.20 | 0.921009324 | Allantoate |
| 3PG | 90.8% | 40.3% | 81.5% | 0.78 | 0.71 | 0.08 | 0.78 | 1.22 | 2544 | 38.57% | 0.28 | -27.36186722 | 3PG |
| N-Acetylglutamate | 36.3% | 14.3% | 33.4% | 2.96 | 1.07 | 2.21 | 2.79 | 3.52 | 1 | 0.02% | 0.76 | 0.8436219 | N-Acetylglutamate |
| o-Hydroxyhippurate | 669.8% | 16.6% | 669.6% | 2.59 | 17.34 | 0.36 | 0.67 | 1.20 | 1065 | 16.15% | 0.10 | 0.077842605 | o-Hydroxyhippurate |
| Gluconate | 35.8% | 18.9% | 30.9% | 63.70 | 22.77 | 47.79 | 61.35 | 76.65 | 40 | 0.61% | 7.70 | 0.837011807 | Gluconate |
| 4-Hydroxy-3-methoxymandelate | 35.3% | 17.2% | 31.2% | 4.47 | 1.58 | 3.38 | 4.26 | 5.34 | 11 | 0.17% | 0.45 | 0.693203924 | 4-Hydroxy-3-methoxymandelate |
| Saccharate | 49.1% | 14.3% | 47.1% | 5.12 | 2.51 | 3.95 | 4.77 | 5.75 | 2 | 0.03% | 1.25 | 0.887318602 | Saccharate |
| Pantothenate | 99.7% | 18.5% | 98.0% | 3.05 | 3.04 | 1.78 | 2.57 | 3.57 | 45 | 0.68% | 0.21 | 0.980165482 | Pantothenate |
| Biotin | 225.4% | 149.6% | 173.3% | 1.10 | 2.47 | 0.07 | 0.07 | 1.31 | 3493 | 52.96% | 0.13 | 0.961260835 | Biotin |
| N-Acetylneuraminate | 21.6% | 9.0% | 19.7% | 18.19 | 3.94 | 15.62 | 17.98 | 20.49 | 14 | 0.21% | 1.67 | 0.337588803 | N-Acetylneuraminate |
| Mean for anions | 81.0% | 23.7% | 75.8% | 33.1 | 19.0 | 20.4 | 29.5 | 41.6 | 326 | | 3.0 | -3.1 | |
| Median for anions | 61.4% | 19.6% | 55.1% | 5.1 | 3.2 | 3.7 | 4.7 | 5.9 | 19 | | 0.8 | 0.8 | |

CV: coefficient of variation, ICC: intraclass correlation coefficient, ND: not detectable values, QC: quality control, SD: standard deviation

*ICC was estimated by following; 1 - (Total CV of QC samples )^2 / (Total CV of subject samples )^2

**Table S4. Statistical summary of metabolites measured in spot and 24-hours samples.**

| | | spot-24hr samples | | QC-participants samples | |
|---|---|---|---|---|---|
| **Metabolite name** | **Pearson's correlation** | **spot_CV** | **24hr_CV** | **QC CV** | **participants CV** |
| **Cations** | | | | | |
| Gly | 0.7601549 | 58.7% | 58.5% | 5.6% | 69.2% |
| Trimethylamine N-oxide | 0.7608579 | 96.2% | 115.7% | 10.7% | 115.4% |
| beta-Ala | 0.2446333 | 86.8% | 53.9% | 17.9% | 89.2% |
| Ala | 0.6799797 | 44.4% | 42.4% | 6.1% | 69.7% |
| 3-Aminoisobutyrate | 0.9843111 | 121.2% | 129.6% | 6.5% | 114.2% |
| 2AB | 0.6389092 | 32.9% | 37.6% | 19.4% | 82.5% |
| N,N-Dimethylglycine | 0.8588088 | 63.5% | 68.0% | 9.3% | 326.5% |
| Choline | 0.62655 | 27.1% | 46.3% | 12.7% | 49.8% |
| Ser | 0.7463013 | 43.5% | 37.5% | 5.8% | 54.4% |
| Pro | 0.593298 | 37.0% | 38.7% | 18.1% | 145.6% |
| Guanidinoacetate | 0.8851773 | 48.0% | 45.6% | 6.4% | 50.3% |
| Val | 0.7264474 | 24.7% | 30.1% | 15.5% | 287.9% |
| Betaine | 0.8688857 | 73.4% | 71.8% | 11.1% | 149.0% |
| Thr | 0.62649 | 59.2% | 54.4% | 5.1% | 95.2% |
| Taurine | 0.6959262 | 62.7% | 64.3% | 12.1% | 70.0% |
| Creatine | 0.807713 | 114.4% | 120.1% | 5.5% | 154.4% |
| Ile | 0.5882272 | 28.0% | 41.4% | 28.7% | 1387.2% |
| Leu | 0.6520756 | 26.8% | 34.0% | 11.1% | 365.4% |
| Asn | 0.7460096 | 63.2% | 47.7% | 9.1% | 81.2% |
| Ornithine | 0.6874877 | 52.3% | 68.9% | 14.6% | 179.0% |
| Asp | 0.2295697 | 24.3% | 38.4% | 134.8% | 707.9% |
| Hypoxanthine | 0.1256587 | 42.3% | 51.4% | 7.0% | 49.9% |
| 1-Methylnicotinamide | 0.9138651 | 42.7% | 44.7% | 16.0% | 46.5% |
| Trigonelline | 0.8721525 | 70.7% | 89.6% | 11.3% | 93.6% |
| Urocanate | NA | 34.6% | 17.8% | 26.5% | 232.7% |
| Proline betaine | 0.8673132 | 84.7% | 87.2% | 11.5% | 146.7% |
| gamma-Butyrobetaine | 0.5847118 | 61.9% | 62.4% | 21.7% | 113.8% |
| Gln | 0.7388563 | 37.1% | 42.2% | 5.9% | 84.2% |
| Lys | 0.9522121 | 126.5% | 172.1% | 5.7% | 141.5% |
| Glu | 0.6380802 | 26.5% | 38.5% | 15.8% | 64.7% |
| Met | 0.5745359 | 23.3% | 21.7% | 27.7% | 180.7% |
| His | 0.7715773 | 45.4% | 47.9% | 6.5% | 55.5% |
| alpha-Aminoadipate | 0.6358968 | 37.1% | 70.6% | 8.3% | 119.4% |
| Carnitine | 0.5498849 | 62.3% | 62.3% | 12.1% | 94.6% |
| Phe | 0.7970296 | 28.1% | 26.9% | 7.0% | 96.1% |
| 3-Methylhistidine | 0.8061715 | 52.8% | 70.3% | 5.4% | 88.6% |
| Arg | 0.6955457 | 38.7% | 45.3% | 12.7% | 219.8% |
| Guanidinosuccinate | 0.9523849 | 30.0% | 33.5% | 6.6% | 49.0% |
| Indole-3-acetate(urine) | NA | NA | NA | 29.6% | 69.0% |
| Citrulline | 0.4650878 | 45.9% | 54.0% | 30.1% | 222.7% |
| Tyr | 0.7739906 | 35.5% | 35.5% | 6.2% | 87.1% |
| SDMA | 0.8990669 | 20.6% | 22.1% | 9.4% | 21.3% |
| ADMA | 0.8936741 | 19.2% | 20.9% | 8.9% | 24.0% |
| o-Acetylcarnitine | 0.7902537 | 95.5% | 176.5% | 10.7% | 132.3% |
| Trp | 0.9036947 | 36.1% | 35.4% | 6.1% | 50.7% |
| Cystine | 0.9460472 | 84.2% | 116.0% | 7.2% | 105.5% |
| Uridine | 0.6388412 | 10.5% | 10.2% | 12.3% | 18.5% |
| Adenosine | 0.8261426 | 25.5% | 27.1% | 19.6% | 33.2% |
| Ethanolamine | 0.8703094 | 24.6% | 24.3% | 9.5% | 26.3% |
| Piperidine | 0.8661732 | 107.0% | 104.8% | 9.3% | 159.4% |
| 1-Methylhistamine | 0.6716862 | 22.2% | 33.1% | 29.8% | 44.7% |
| Imidazole-4-acetate | 0.7184761 | 38.0% | 37.1% | 16.7% | 70.4% |
| N-Acetylputrescine | 0.9320704 | 29.0% | 33.9% | 13.2% | 33.4% |
| 5-Aminolevulinate | 0.8626726 | 32.5% | 28.7% | 16.2% | 30.1% |
| gamma-Guanidinobutyrate | 0.9200355 | 87.3% | 103.4% | 15.9% | 142.9% |
| 4-(beta-Acetylaminoethyl)imidazole | 0.687267 | 71.3% | 74.9% | 33.8% | 236.2% |
| Allantoin | 0.01632789 | 19.6% | 21.2% | 62.7% | 53.3% |
| 7-Methylguanine | 0.6936763 | 24.2% | 24.3% | 11.4% | 32.3% |
| N1-Acetylspermidine | 0.9338291 | 35.9% | 41.6% | 24.1% | 45.7% |
| N8-Acetylspermidine | 0.7783632 | 20.4% | 21.9% | 22.3% | 35.7% |
| Gly-Leu | 0.9702783 | 54.7% | 57.7% | 12.7% | 35.9% |
| N-epsilon-Acetyllysine | 0.7173509 | 25.5% | 26.4% | 10.4% | 28.8% |
| N6,N6,N6-Trimethyllysine | 0.3106524 | 25.9% | 35.9% | 8.7% | 45.1% |
| N-Acetylhistidine | 0.8296907 | 33.1% | 35.5% | 16.7% | 37.9% |
| SAM+ | 0.6527813 | 22.9% | 23.2% | 15.5% | 29.4% |
| N-Acetylglucosamine | 0.6995127 | 16.7% | 14.7% | 17.0% | 30.6% |
| Cystathionine | 0.4643321 | 34.8% | 45.4% | 62.9% | 808.8% |
| 7,8-Dihydrobiopterin | 0.5015996 | 26.8% | 27.7% | 32.9% | 41.3% |
| 1-Methyladenosine | 0.9528463 | 25.7% | 25.7% | 14.7% | 25.3% |
| Mean for cations | 0.717468957 | 46.8% | 52.5% | 17.0% | 134.5% |
| Median for cations | 0.7460096 | 37.0% | 41.9% | 12.1% | 81.2% |
| **Anions** | | | | | |
| Lactate | -0.03568166 | 53.1% | 61.8% | 26.6% | 73.7% |
| 3-Hydroxypropionate | NA | 22.2% | 30.5% | NA | NA |
| Malonate | 0.4231227 | 31.1% | 31.3% | 593.3% | 63.1% |
| 3-Hydroxybutyrate | 0.7613301 | 51.8% | 54.4% | 11.9% | 158.5% |
| 4-Oxopentanoate(urine) | NA | NA | NA | 23.2% | 38.9% |
| Succinate | 0.843445 | 40.5% | 41.9% | 17.7% | 56.0% |
| 2-Hydroxypentanoate | 0.9188988 | 46.6% | 50.1% | 15.0% | 50.1% |
| Isethionate | 0.9659622 | 37.9% | 31.9% | 10.8% | 26.5% |
| 5-Oxoproline | 0.6805983 | 22.0% | 18.9% | 11.5% | 25.4% |
| 4-Methyl-2-oxopentanoate | 0.5405472 | 41.8% | 39.1% | 20.3% | 46.6% |
| Glutarate | 0.7517839 | 37.0% | 43.8% | 52.0% | 75.4% |
| 3-Ureidopropionate | NA | NA | 8.2% | NA | NA |
| Malate | 0.7324206 | 91.4% | 80.2% | 21.2% | 234.1% |
| Threonate | 0.8118612 | 14.9% | 15.2% | 13.2% | 26.2% |
| Ethanolamine phosphate(urine) | NA | NA | NA | 15.4% | 55.8% |
| 2-Oxoglutarate | 0.8732833 | 62.4% | 61.1% | 69.7% | 75.8% |
| Pimelate | 0.7284129 | 62.7% | 90.0% | 12.8% | 65.7% |
| Urate(urine) | NA | NA | NA | 13.2% | 41.8% |
| Glycerophosphate | 0.6220944 | 47.9% | 80.4% | 13.8% | 61.4% |
| trans-Aconitate | 0.678751 | 70.8% | 21.1% | 19.2% | 44.7% |
| cis-Aconitate(urine) | NA | NA | NA | 15.7% | 30.9% |
| N-Acetylaspartate | 0.9621174 | 43.1% | 42.7% | 17.1% | 39.0% |
| Hippurate(urine) | NA | NA | NA | 14.6% | 94.2% |
| Homovanillate(urine) | NA | NA | NA | 21.0% | 93.3% |
| 4-Pyridoxate(urine) | NA | NA | NA | 22.2% | 130.2% |
| Azelate | 0.03671562 | 49.3% | 59.4% | 18.0% | 76.0% |
| Isocitrate | 0.8511513 | 20.1% | 22.4% | 11.8% | 25.0% |
| Citrate | 0.8261496 | 47.7% | 50.2% | 16.3% | 54.8% |
| Quinate(urine) | NA | NA | NA | 12.2% | 106.9% |
| Glucuronate(urine) | NA | NA | NA | 16.1% | 34.0% |
| Cysteine S-sulfate | 0.2698143 | 28.6% | 34.2% | 47.5% | 48.7% |
| Mucate | 0.809932 | 37.3% | 44.8% | 19.4% | 68.3% |
| 3-Indoxyl sulfate | 0.8445783 | 69.5% | 73.3% | 13.1% | 67.0% |
| Glycolate(urine) | NA | NA | NA | 21.3% | 40.9% |
| Oxamate(urine) | NA | NA | NA | 21.2% | 89.0% |
| 3-Hydroxypropionate(urine) | NA | 13.1% | 11.7% | 361.2% | 64.3% |
| 2-Hydroxyisobutyrate | 0.5694503 | 23.4% | 23.7% | 14.3% | 33.4% |
| Itaconate(urine) | NA | NA | NA | 792.9% | 54.9% |
| N-Acetyl-beta-alanine(urine) | NA | NA | NA | 16.0% | 61.3% |
| 3-Ureidopropionate(urine) | NA | NA | NA | 69.1% | 117.2% |
| Adipate | 0.7970837 | 45.2% | 71.7% | 14.8% | 55.2% |
| p-Hydroxyphenylacetate(urine) | NA | NA | NA | 20.5% | 92.2% |
| 3-Hydroxy-3-methylglutarate | 0.8639339 | 27.7% | 29.8% | 21.9% | 35.7% |
| 2,3-Pyridinedicarboxylate | 0.8662418 | 29.0% | 29.4% | 19.0% | 38.4% |
| 4-Hydroxymandelate(urine) | NA | NA | NA | 26.8% | 41.3% |
| Suberate | 0.1535246 | 41.0% | 69.8% | 16.3% | 69.2% |
| Allantoate(urine) | NA | NA | NA | 33.4% | 92.1% |
| 3PG | 0.3774146 | 39.8% | 51.1% | 483.4% | 46.8% |
| N-Acetylglutamate | 0.7987997 | 34.9% | 34.6% | 14.3% | 36.6% |
| o-Hydroxyhippurate | 0.8685944 | 110.0% | 125.8% | 643.2% | 617.1% |
| Gluconate(urine) | NA | NA | NA | 14.4% | 35.5% |
| 4-Hydroxy-3-methoxymandelate(urine) | | NA | NA | 19.6% | 35.4% |
| Saccharate | 0.9119281 | 35.3% | 34.9% | 16.5% | 49.0% |
| Pantothenate(urine) | NA | NA | NA | 14.0% | 99.5% |
| Biotin(urine) | NA | NA | NA | 44.4% | 133.1% |
| N-Acetylneuraminate | 0.8693421 | 20.7% | 17.7% | 17.6% | 21.2% |
| Mean for anions | 0.686675052 | 42.6% | 45.3% | 72.6% | 74.9% |
| Median for anions | 0.7979417 | 40.1% | 41.9% | 17.8% | 55.5% |

Ascorbic acid was added to these samples during pretreatment, and the metabolites detected were different from the entire cohort.

**Table S5. Concentrations of metabolite stratified with sex, age and lifestyle.**

| | All ages Mean (μmol/L) | -44y Mean (μmol/L) | 45-54y Mean (μmol/L) | 55-y Mean (μmol/L) | Coefficients (per year) | 95% CI upper | 95% CI lower |
|---|---|---|---|---|---|---|---|
| N | 32 | 12 | 9 | 11 | | | |
| Ethanolamine_24hours | 23.31±5.86 | 24.67±6.21 | 22.44±3.91 | 23.2±6.96 | -1.075 | -3.58 | 1.43 |
| Ethanolamine_spot | 25.28±6.22 | 26.83±4.51 | 24.56±3.81 | 24.9±9.16 | -1.337 | -3.98 | 1.31 |
| Gly_24hours | 87.5±52.26 | 109.92±65.73 | 90.67±47.02 | 62.8±22.79 | -24.664 | -45.35 | -3.98 |
| Gly_spot | 67.03±39.95 | 77.67±42.98 | 75.67±42.09 | 50.6±30.85 | -14.497 | -30.93 | 1.94 |
| Ala_24hours | 27.09±12.2 | 29.67±16.01 | 23.56±8.57 | 28.1±10.09 | -1.302 | -6.56 | 3.96 |
| Ala_spot | 19.86±8.58 | 20.67±8.44 | 18.06±8.92 | 21.1±9.3 | -0.137 | -3.86 | 3.58 |
| N,N-Dimethylglycine_24hours | 7.56±4.88 | 7.5±3.86 | 8.74±7.93 | 6.81±2.31 | -0.398 | -2.51 | 1.71 |
| N,N-Dimethylglycine_spot | 5.41±3.71 | 4.84±1.86 | 6.61±6.04 | 5.23±2.89 | 0.127 | -1.48 | 1.73 |
| 3-Aminoisobutyrate_24hours | 55.5±68.32 | 49.38±56.11 | 70.39±69.71 | 53.2±86.45 | 0.566 | -29.03 | 30.16 |
| 3-Aminoisobutyrate_spot | 60.55±79.76 | 53.89±62.63 | 69.82±69.3 | 64.86±111.53 | 3.329 | -31.20 | 37.86 |
| Ser_24hours | 39.25±17.37 | 47.58±23.78 | 35.44±9.29 | 34.5±10.12 | -7.216 | -14.25 | -0.19 |
| Ser_spot | 31.09±11.91 | 34.75±11.4 | 29.56±9.65 | 29.6±14.12 | -3.218 | -8.24 | 1.80 |
| Guanidinoacetate_24hours | 62.97±30.84 | 65.92±33.78 | 65.44±32.63 | 59.4±29.12 | -4.050 | -17.32 | 9.22 |
| Guanidinoacetate_spot | 50.5±23.47 | 50.58±23.74 | 54±22.35 | 49.5±26.36 | -1.459 | -11.61 | 8.69 |
| Thr_24hours | 19.83±11.94 | 25.39±16.58 | 17±6.24 | 16.71±6.94 | -4.710 | -9.57 | 0.15 |
| Thr_spot | 15.38±8.55 | 17.25±6.81 | 14.12±6.51 | 15.19±11.9 | -1.459 | -5.12 | 2.20 |
| Creatine_24hours | 131±152.45 | 133.13±137.17 | 173.39±227.61 | 100.9±83.36 | -18.834 | -84.50 | 46.83 |
| Creatine_spot | 50.83±62 | 35.98±43.19 | 69.56±83.39 | 55.93±62.58 | 8.191 | -18.49 | 34.87 |
| Asn_24hours | 15.96±10.23 | 20.92±14.93 | 13.23±3.75 | 13.42±4.29 | -4.112 | -8.27 | 0.05 |
| Asn_spot | 11.24±5.47 | 12.58±5.72 | 10.5±4.31 | 11.03±6.16 | -1.100 | -3.43 | 1.24 |
| Hypoxanthine_24hours | 7.22±3.09 | 7.34±3.92 | 7.33±2.22 | 6.82±3.04 | -0.178 | -1.51 | 1.16 |
| Hypoxanthine_spot | 14.3±7.44 | 13.16±3.08 | 12.24±5.02 | 17.26±11.85 | 2.003 | -1.13 | 5.14 |
| Gln_24hours | 75.97±28.65 | 94.33±33.76 | 68.67±20.21 | 63.9±16.64 | -16.328 | -27.14 | -5.52 |
| Gln_spot | 62.94±26.91 | 72.75±22.7 | 62.11±29.88 | 55.7±26.89 | -9.929 | -20.98 | 1.12 |
| Lys_24hours | 59.02±75.69 | 89±116.26 | 42.06±29.6 | 42.1±21.71 | -24.685 | -56.16 | 6.79 |
| Lys_spot | 45.85±80.27 | 67.92±127.54 | 33.16±26.43 | 34.18±24.49 | -18.083 | -52.19 | 16.03 |
| His_24hours | 104.59±48.32 | 134.67±55.71 | 93.67±41.42 | 82.6±25.69 | -27.141 | -45.47 | -8.82 |
| His_spot | 83.53±40.66 | 101.17±33.99 | 78.44±42.25 | 71.5±43.16 | -16.434 | -32.94 | 0.08 |
| alpha-Aminoadipate_24hours | 5.13±1.93 | 5.55±2.05 | 4.43±1.87 | 5.4±1.85 | -0.173 | -1.00 | 0.66 |
| alpha-Aminoadipate_spot | 3.81±2.67 | 3.73±1.22 | 3.13±1.01 | 4.67±4.51 | 0.349 | -0.80 | 1.50 |
| Phe_24hours | 7.85±2.22 | 8.58±2.67 | 7.49±2.36 | 7.48±1.39 | -0.629 | -1.56 | 0.30 |
| Phe_spot | 6.9±1.87 | 7.38±2 | 6.57±1.91 | 6.87±1.7 | -0.366 | -1.17 | 0.43 |
| 3.Methylhistidine_24hours | 118.25±63.51 | 126.75±74.05 | 102.78±55.02 | 125.5±62.99 | -2.819 | -30.31 | 24.67 |
| 3.Methylhistidine_spot | 97±69.36 | 103.42±66.91 | 74.89±32.51 | 111.3±96.63 | 1.959 | -28.08 | 31.99 |
| Guanidinosuccinate_24hours | 5.49±1.72 | 5.46±2.15 | 4.8±1.48 | 6.19±1.23 | 0.309 | -0.43 | 1.04 |
| Guanidinosuccinate_spot | 5.47±1.86 | 5.4±2.02 | 4.48±1.55 | 6.51±1.6 | 0.460 | -0.33 | 1.25 |
| Tyr_24hours | 14.18±5.18 | 16.06±6.33 | 13.9±4.87 | 12.6±3.58 | -1.851 | -3.99 | 0.28 |
| Tyr_spot | 12.95±4.67 | 13.99±4.6 | 13.46±4.88 | 11.76±4.69 | -1.287 | -3.25 | 0.68 |
| N6,N6,N6,Trimethyllysine_24hours | 13.39±3.52 | 13.55±3.38 | 12.83±4.93 | 13.64±2.6 | 0.052 | -1.47 | 1.58 |
| N6,N6,N6,Trimethyllysine_spot | 11.98±4.45 | 10.68±2.92 | 11.22±3.58 | 14.1±6.21 | 1.645 | -0.18 | 3.47 |
| ADMA_24hours | 7.75±1.5 | 7.83±1.33 | 7.33±1.09 | 8.23±1.94 | 0.089 | -0.56 | 0.74 |
| ADMA_spot | 7.31±1.55 | 7.15±1.08 | 7.07±1.01 | 7.92±2.25 | 0.266 | -0.40 | 0.93 |
| SDMA_24hours | 7.88±1.6 | 7.83±1.26 | 7.96±1.77 | 7.97±1.99 | 0.012 | -0.68 | 0.71 |
| SDMA_spot | 8.12±1.81 | 7.86±1.41 | 8.04±1.6 | 8.58±2.48 | 0.297 | -0.48 | 1.07 |
| Trp_24hours | 10.77±3.89 | 12.6±4.87 | 9.79±2.68 | 9.79±3 | -1.530 | -3.11 | 0.06 |
| Trp_spot | 10.8±3.84 | 12.23±4.28 | 10.21±2.87 | 10.01±3.95 | -1.262 | -2.86 | 0.33 |
| Cystine_24hours | 13.7±11.75 | 19.03±17.96 | 11.14±4.07 | 10.24±2.08 | -4.563 | -9.36 | 0.23 |
| Cystine_spot | 11.18±13.1 | 14.92±20.72 | 9.46±4.55 | 8.74±4.1 | -3.236 | -8.78 | 2.31 |

| | male Mean (μmol/L) | female Mean (μmol/L) | Coefficients (female) | 95% CI upper | 95% CI lower |
|---|---|---|---|---|---|
| N | 17 | 15 | | | |
| Ethanolamine_24hours | 20.82±5.56 | 26.13±4.97 | 5.310 | 1.48 | 9.14 |
| Ethanolamine_spot | 22.29±5.51 | 28.67±5.27 | 6.373 | 2.47 | 10.28 |
| Gly_24hours | 61.41±44.61 | 117.07±44.84 | 55.655 | 23.31 | 88.00 |
| Gly_spot | 46.94±32.13 | 89.8±36.17 | 42.859 | 18.21 | 67.51 |
| Ala_24hours | 26.71±15.67 | 27.53±6.95 | 0.827 | -8.14 | 9.79 |
| Ala_spot | 20.15±9.01 | 19.53±8.37 | -0.614 | -6.92 | 5.70 |
| N,N-Dimethylglycine_24hours | 7.45±5.9 | 7.69±3.6 | 0.246 | -3.34 | 3.84 |
| N,N-Dimethylglycine_spot | 5.34±4.59 | 5.49±2.53 | 0.152 | -2.58 | 2.88 |
| 3-Aminoisobutyrate_24hours | 25.25±32.21 | 89.78±82.33 | 64.527 | 20.42 | 108.63 |
| 3-Aminoisobutyrate_spot | 26.35±36.17 | 99.32±97.77 | 72.973 | 21.01 | 124.94 |
| Ser_24hours | 37.53±21.98 | 41.2±10.42 | 3.671 | -9.03 | 16.37 |
| Ser_spot | 29.24±12.29 | 33.2±11.53 | 3.965 | -4.67 | 12.60 |
| Guanidinoacetate_24hours | 46.24±22.1 | 81.93±28.67 | 35.698 | 17.34 | 54.06 |
| Guanidinoacetate_spot | 34.88±14.49 | 68.2±18.65 | 33.318 | 21.34 | 45.30 |
| Thr_24hours | 19.32±15.29 | 20.4±6.91 | 1.082 | -7.69 | 9.85 |
| Thr_spot | 14.38±7.82 | 16.51±9.45 | 2.131 | -4.11 | 8.37 |
| Creatine_24hours | 41.77±64.85 | 232.13±161.34 | 190.363 | 103.58 | 277.15 |
| Creatine_spot | 14.65±23.18 | 91.85±67.11 | 77.200 | 41.84 | 112.56 |
| Asn_24hours | 15.74±13.31 | 16.21±5.4 | 0.465 | -7.06 | 7.99 |
| Asn_spot | 10.76±6.2 | 11.79±4.68 | 1.022 | -2.98 | 5.03 |
| Hypoxanthine_24hours | 7.18±3.9 | 7.26±1.91 | 0.084 | -2.19 | 2.35 |
| Hypoxanthine_spot | 14.97±9.09 | 13.55±5.21 | -1.424 | -6.87 | 4.02 |
| Gln_24hours | 69.94±31.72 | 82.8±23.94 | 12.859 | -7.65 | 33.37 |
| Gln_spot | 59.59±29.69 | 66.73±23.81 | 7.145 | -12.46 | 26.75 |
| Lys_24hours | 42.91±24.99 | 77.27±106.31 | 34.367 | -19.81 | 88.54 |
| Lys_spot | 31.94±24.73 | 61.61±114.3 | 29.672 | -28.31 | 87.66 |
| His_24hours | 98.35±52.85 | 111.67±43.34 | 13.314 | -21.88 | 48.50 |
| His_spot | 79.82±42.27 | 87.73±39.77 | 7.910 | -21.84 | 37.66 |
| alpha-Aminoadipate_24hours | 4.56±1.87 | 5.77±1.85 | 1.208 | -0.14 | 2.55 |
| alpha-Aminoadipate_spot | 3.2±1.12 | 4.49±3.67 | 1.293 | -0.61 | 3.20 |
| Phe_24hours | 7.09±2.21 | 8.7±1.97 | 1.606 | 0.08 | 3.13 |
| Phe_spot | 6.39±1.65 | 7.47±2 | 1.079 | -0.24 | 2.40 |
| 3.Methylhistidine_24hours | 108.24±59.01 | 129.6±68.49 | 21.365 | -24.66 | 67.39 |
| 3.Methylhistidine_spot | 96.41±79.86 | 97.67±57.98 | 1.255 | -49.75 | 52.26 |
| Guanidinosuccinate_24hours | 5.02±1.37 | 6.03±1.95 | 1.016 | -0.19 | 2.22 |
| Guanidinosuccinate_spot | 4.96±1.58 | 6.05±2.03 | 1.088 | -0.22 | 2.40 |
| Tyr_24hours | 13.29±5.83 | 15.19±4.3 | 1.893 | -1.85 | 5.64 |
| Tyr_spot | 12.61±4.7 | 13.33±4.77 | 0.722 | -2.70 | 4.15 |
| N6,N6,N6,Trimethyllysine_24hours | 12.31±3.44 | 14.62±3.3 | 2.314 | -0.13 | 4.76 |
| N6,N6,N6,Trimethyllysine_spot | 11.18±3.2 | 12.88±5.52 | 1.704 | -1.51 | 4.92 |
| ADMA_24hours | 6.93±1.21 | 8.69±1.25 | 1.757 | 0.87 | 2.65 |
| ADMA_spot | 6.49±0.96 | 8.25±1.58 | 1.758 | 0.83 | 2.69 |
| SDMA_24hours | 6.9±0.67 | 8.98±1.65 | 2.080 | 1.19 | 2.97 |
| SDMA_spot | 7.11±0.73 | 9.26±2 | 2.154 | 1.09 | 3.22 |
| Trp_24hours | 10.21±4.27 | 11.4±3.44 | 1.188 | -1.64 | 4.01 |
| Trp_spot | 10.46±4 | 11.19±3.75 | 0.735 | -2.08 | 3.55 |
| Cystine_24hours | 10.85±3.83 | 16.93±16.37 | 6.074 | -2.26 | 14.41 |
| Cystine_spot | 8.69±4.22 | 13.99±18.54 | 5.293 | -4.14 | 14.72 |

| | smoke Mean (μmol/L) | nonsmoke Mean (μmol/L) | Coefficients (smoke) | 95% CI upper | 95% CI lower | drink Mean (μmol/L) | nondrink Mean (μmol/L) | Coefficients (drink) | 95% CI upper | 95% CI lower |
|---|---|---|---|---|---|---|---|---|---|---|
| N | 3 | 29 | | | | 10 | 22 | | | |
| Ethanolamine_24hours | 17.67±1.53 | 23.9±5.84 | -6.230 | -13.23 | 0.77 | 21.6±4.72 | 24.09±6.26 | -2.491 | -7.04 | 2.06 |
| Ethanolamine_spot | 18±4.58 | 26.03±5.92 | -8.034 | -15.27 | -0.80 | 24.6±3.5 | 25.59±7.18 | -0.991 | -5.90 | 3.92 |
| Gly_24hours | 44±15.13 | 92±52.76 | -48.000 | -111.32 | 15.32 | 91.5±59.57 | 85.68±50 | 5.818 | -35.50 | 47.14 |
| Gly_spot | 32.33±18.15 | 70.62±40.01 | -38.287 | -86.51 | 9.94 | 76.9±43.63 | 62.55±38.37 | 14.355 | -16.82 | 45.53 |
| Ala_24hours | 19.33±4.73 | 27.9±12.49 | -8.563 | -2.95 | 0.71 | 20.4±5.32 | 30.14±13.28 | -9.736 | -18.68 | -0.79 |
| Ala_spot | 15.17±6.29 | 20.34±8.73 | -5.178 | -2.50 | 0.49 | 18.2±4.24 | 20.61±9.96 | -2.414 | -9.15 | 4.32 |
| N,N-Dimethylglycine_24hours | 6.53±3.04 | 7.67±5.06 | -1.136 | -23.58 | 6.46 | 6.65±3.42 | 7.98±5.44 | -1.327 | -5.16 | 2.51 |
| N,N-Dimethylglycine_spot | 4.37±1.99 | 5.52±3.85 | -1.154 | -15.81 | 5.46 | 5.48±1.75 | 5.38±4.36 | 0.098 | -2.84 | 3.04 |
| 3-Aminoisobutyrate_24hours | 20.33±10.69 | 59.14±70.8 | -38.805 | -7.27 | 5.00 | 57.86±69.69 | 54.43±69.31 | 3.433 | -50.64 | 57.51 |
| 3-Aminoisobutyrate_spot | 23.47±19.39 | 64.39±82.78 | -40.923 | -5.80 | 3.50 | 55.4±68.56 | 62.9±85.77 | -7.495 | -70.58 | 55.59 |
| Ser_24hours | 26±12.77 | 40.62±17.37 | -14.621 | -123.59 | 45.98 | 39±12.54 | 39.36±19.45 | -0.364 | -14.12 | 13.39 |
| Ser_spot | 22±11.53 | 32.03±11.74 | -10.034 | -140.18 | 58.33 | 34.2±8.02 | 29.68±13.24 | 4.518 | -4.76 | 13.80 |
| Guanidinoacetate_24hours | 40.67±1.15 | 65.28±31.52 | -24.609 | -35.81 | 6.56 | 54.6±38.82 | 66.77±26.63 | -12.173 | -36.16 | 11.82 |
| Guanidinoacetate_spot | 28±8.19 | 52.83±23.35 | -24.828 | -24.56 | 4.49 | 46.3±26.85 | 52.41±22.18 | -6.109 | -24.55 | 12.33 |
| Thr_24hours | 10.83±6.21 | 20.76±12.06 | -9.922 | -62.33 | 13.11 | 19.37±8.24 | 20.03±13.46 | -0.662 | -10.11 | 8.79 |
| Thr_spot | 8.67±5.5 | 16.08±8.57 | -7.409 | -52.89 | 3.23 | 17.62±6.05 | 14.36±9.42 | 3.256 | -3.40 | 9.91 |
| Creatine_24hours | 31.67±37.58 | 141.28±156.41 | -109.613 | -24.49 | 4.64 | 110.05±192.3 | 140.53±134.75 | -30.477 | -150.65 | 89.69 |
| Creatine_spot | 6.2±1.87 | 55.45±63.4 | -49.252 | -17.81 | 2.99 | 43.91±75.62 | 53.98±56.48 | -10.072 | -59.01 | 38.87 |
| Asn_24hours | 10±4 | 16.58±10.52 | -6.576 | -297.16 | 77.93 | 14.71±4.63 | 16.53±12.01 | -1.817 | -9.89 | 6.26 |
| Asn_spot | 7.03±2.81 | 11.68±5.52 | -4.646 | -125.12 | 26.62 | 11.97±3.63 | 10.91±6.18 | 1.056 | -3.26 | 5.37 |
| Hypoxanthine_24hours | 6.27±1.11 | 7.31±3.22 | -1.047 | -19.23 | 6.07 | 6.88±1.47 | 7.37±3.61 | -0.488 | -2.92 | 1.95 |
| Hypoxanthine_spot | 14.17±8.37 | 14.32±7.51 | -0.151 | -11.32 | 2.02 | 11.42±4.25 | 15.61±8.26 | -4.194 | -9.88 | 1.49 |
| Gln_24hours | 48.33±18.15 | 78.83±28.19 | -30.494 | -4.91 | 2.82 | 75.1±21.35 | 76.36±31.87 | -1.264 | -23.94 | 21.41 |
| Gln_spot | 35±20.22 | 65.83±26.08 | -30.828 | -9.52 | 9.22 | 70.8±20.21 | 59.36±29.17 | 11.436 | -9.44 | 32.31 |
| Lys_24hours | 21.13±14.56 | 62.93±78.48 | -41.801 | -64.72 | 3.73 | 40.61±31.59 | 67.38±88.29 | -26.772 | -85.87 | 32.32 |
| Lys_spot | 12.07±6.9 | 49.34±83.63 | -37.278 | -62.70 | 1.04 | 39.42±30.49 | 48.77±95.31 | -9.353 | -72.81 | 54.10 |
| His_24hours | 67±35.68 | 108.48±48.24 | -41.483 | -135.82 | 52.22 | 95±33.16 | 108.95±53.96 | -13.955 | -51.86 | 23.95 |
| His_spot | 47.67±31.39 | 87.24±40.09 | -39.575 | -137.38 | 62.82 | 86.9±29.43 | 82±45.4 | 4.900 | -27.24 | 37.04 |
| alpha-Aminoadipate_24hours | 4.4±1.93 | 5.2±1.94 | -0.800 | -100.33 | 17.36 | 3.9±1.61 | 5.68±1.83 | -1.782 | -3.15 | -0.41 |
| alpha-Aminoadipate_spot | 2.83±1.24 | 3.91±2.77 | -1.074 | -88.59 | 9.44 | 3.02±0.7 | 4.16±3.15 | -1.144 | -3.22 | 0.93 |
| Phe_24hours | 5.93±2.29 | 8.04±2.16 | -2.111 | -3.21 | 1.61 | 6.91±1.53 | 8.27±2.38 | -1.363 | -3.05 | 0.32 |
| Phe_spot | 5.9±2.42 | 7±1.83 | -1.103 | -4.42 | 2.27 | 6.4±1.08 | 7.13±2.12 | -0.727 | -2.18 | 0.73 |
| 3.Methylhistidine_24hours | 164±99.02 | 113.52±59.31 | 50.483 | -4.80 | 0.57 | 84.4±38.19 | 133.64±67.34 | -49.236 | -96.05 | -2.42 |
| 3.Methylhistidine_spot | 166±148.58 | 89.86±56.45 | 76.138 | -3.42 | 1.22 | 70.2±39.08 | 109.18±77.13 | -38.982 | -91.94 | 13.97 |
| Guanidinosuccinate_24hours | 4.8±1.39 | 5.57±1.75 | -0.766 | -27.23 | 128.20 | 4.54±1.03 | 5.93±1.81 | -1.387 | -2.64 | -0.13 |
| Guanidinosuccinate_spot | 4.73±2.08 | 5.54±1.86 | -0.811 | -6.44 | 158.72 | 4.24±0.9 | 6.03±1.93 | -1.787 | -3.10 | -0.47 |
| Tyr_24hours | 9.3±2.14 | 14.69±5.15 | -5.386 | -2.91 | 1.38 | 12.89±3.64 | 14.77±5.73 | -1.878 | -5.92 | 2.16 |
| Tyr_spot | 8.7±1.76 | 13.39±4.67 | -4.690 | -3.14 | 1.51 | 13.06±3.57 | 12.9±5.17 | 0.160 | -3.54 | 3.86 |
| N6,N6,N6,Trimethyllysine_24hours | 11.33±1.53 | 13.6±3.61 | -2.270 | -11.59 | 0.82 | 11.41±3.15 | 14.29±3.37 | -2.881 | -5.45 | -0.31 |
| N6,N6,N6,Trimethyllysine_spot | 12.53±5.64 | 11.92±4.43 | 0.616 | -10.31 | 0.93 | 10.69±3.58 | 12.56±4.75 | -1.869 | -5.32 | 1.59 |
| ADMA_24hours | 6.77±0.35 | 7.86±1.54 | -1.089 | -6.62 | 2.08 | 7.59±1.36 | 7.83±1.59 | -0.237 | -1.42 | 0.95 |
| ADMA_spot | 6±0.35 | 7.45±1.57 | -1.448 | -4.98 | 6.22 | 7.34±1.04 | 7.3±1.76 | 0.040 | -1.19 | 1.27 |
| SDMA_24hours | 6.77±0.5 | 7.99±1.64 | -1.223 | -2.94 | 0.76 | 7.84±1.59 | 7.89±1.64 | -0.051 | -1.32 | 1.22 |
| SDMA_spot | 6.9±0.7 | 8.24±1.85 | -1.341 | -3.32 | 0.43 | 7.92±1.36 | 8.2±2 | -0.285 | -1.71 | 1.14 |
| Trp_24hours | 8.37±2.81 | 11.02±3.94 | -2.651 | -3.19 | 0.74 | 9.6±2.22 | 11.3±4.39 | -1.700 | -4.71 | 1.31 |
| Trp_spot | 8.57±2.74 | 11.03±3.9 | -2.468 | -3.56 | 0.88 | 10.53±2.24 | 10.93±4.42 | -0.397 | -3.43 | 2.64 |
| Cystine_24hours | 7.7±2.07 | 14.32±12.18 | -6.621 | -7.45 | 2.15 | 10.17±4.77 | 15.3±13.61 | -5.135 | -14.24 | 3.97 |
| Cystine_spot | 5.33±1.21 | 11.78±13.63 | -6.446 | -7.22 | 2.28 | 9.42±4.86 | 11.97±15.53 | -2.553 | -12.88 | 7.78 |
| | | | | -21.21 | 7.96 | | | | | |
| | | | | -22.765796 | 9.873842 | | | | | |

These metabolites was selected by CV less than 10% in the QC sample. Piperidine was too sparse to be compared between the groups in these sample because ascorbic acid was added in the pretreatment.

# S Fig1

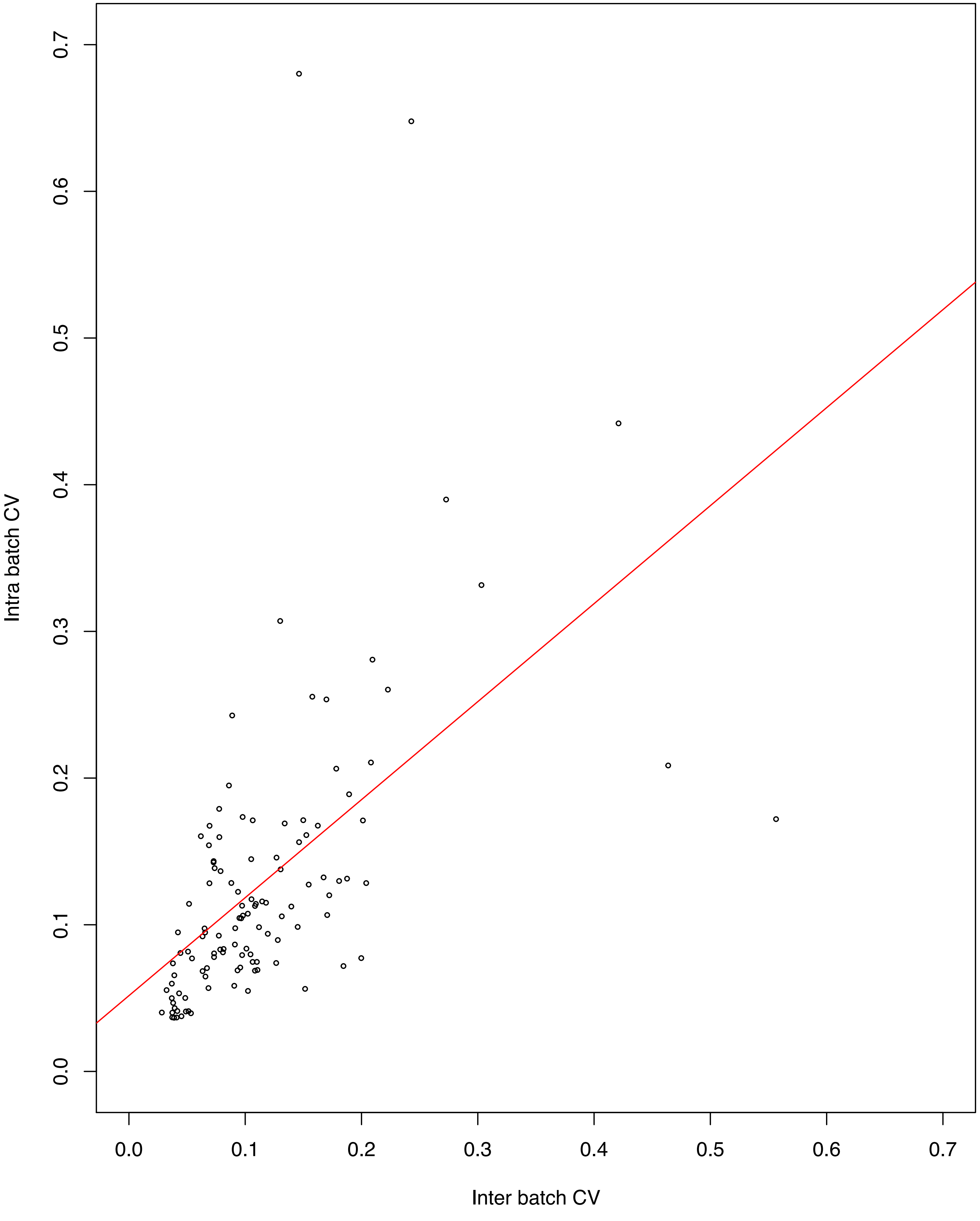


R Core Team (2018). R: A language and environment for statistical computing. R Foundation for Statistical Computing, Vienna, Austria. URL https://www.R-project.org/.

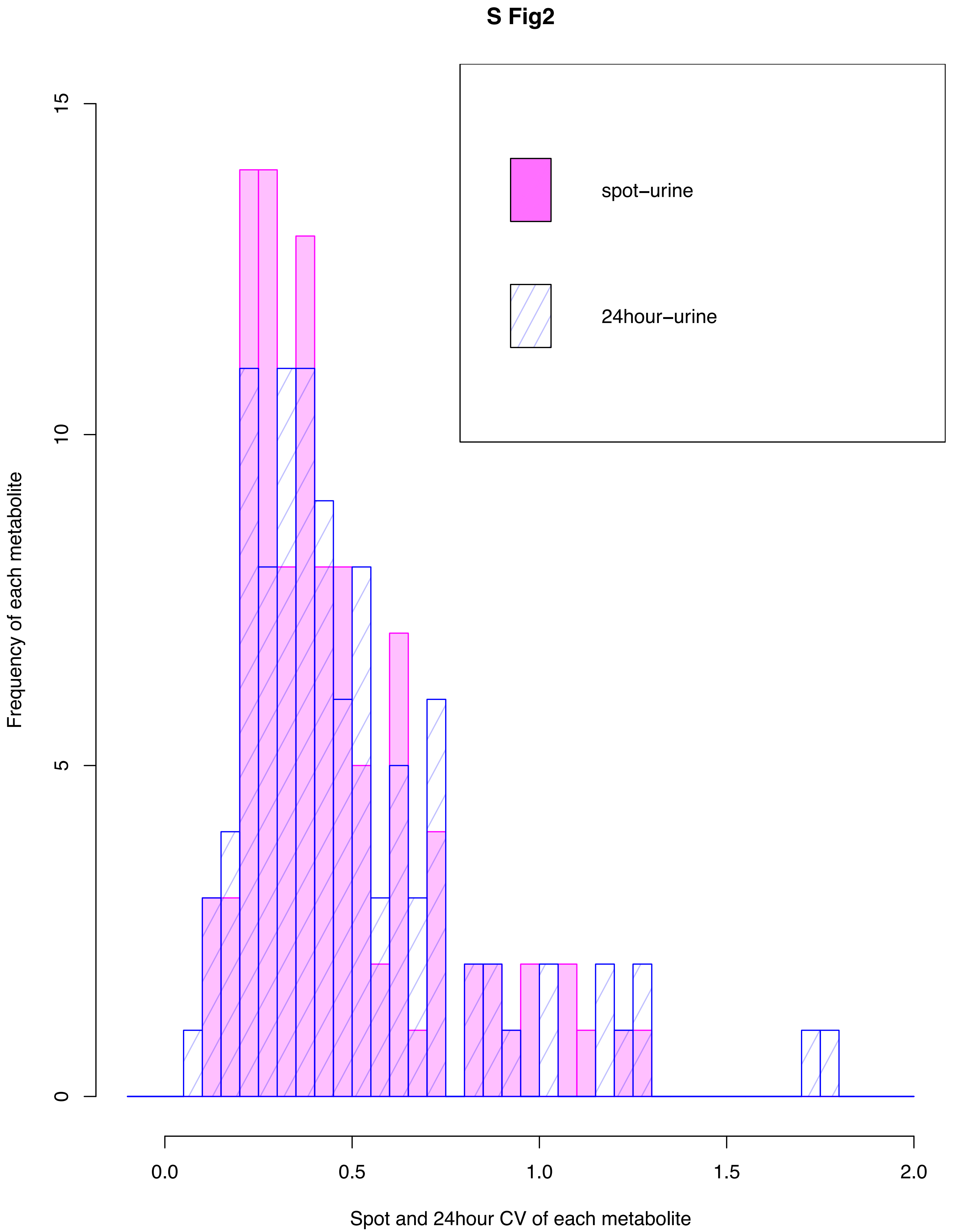


R Core Team (2018). R: A language and environment for statistical computing. R Foundation for Statistical Computing, Vienna, Austria. URL https://www.R-project.org/.

## SFig 3

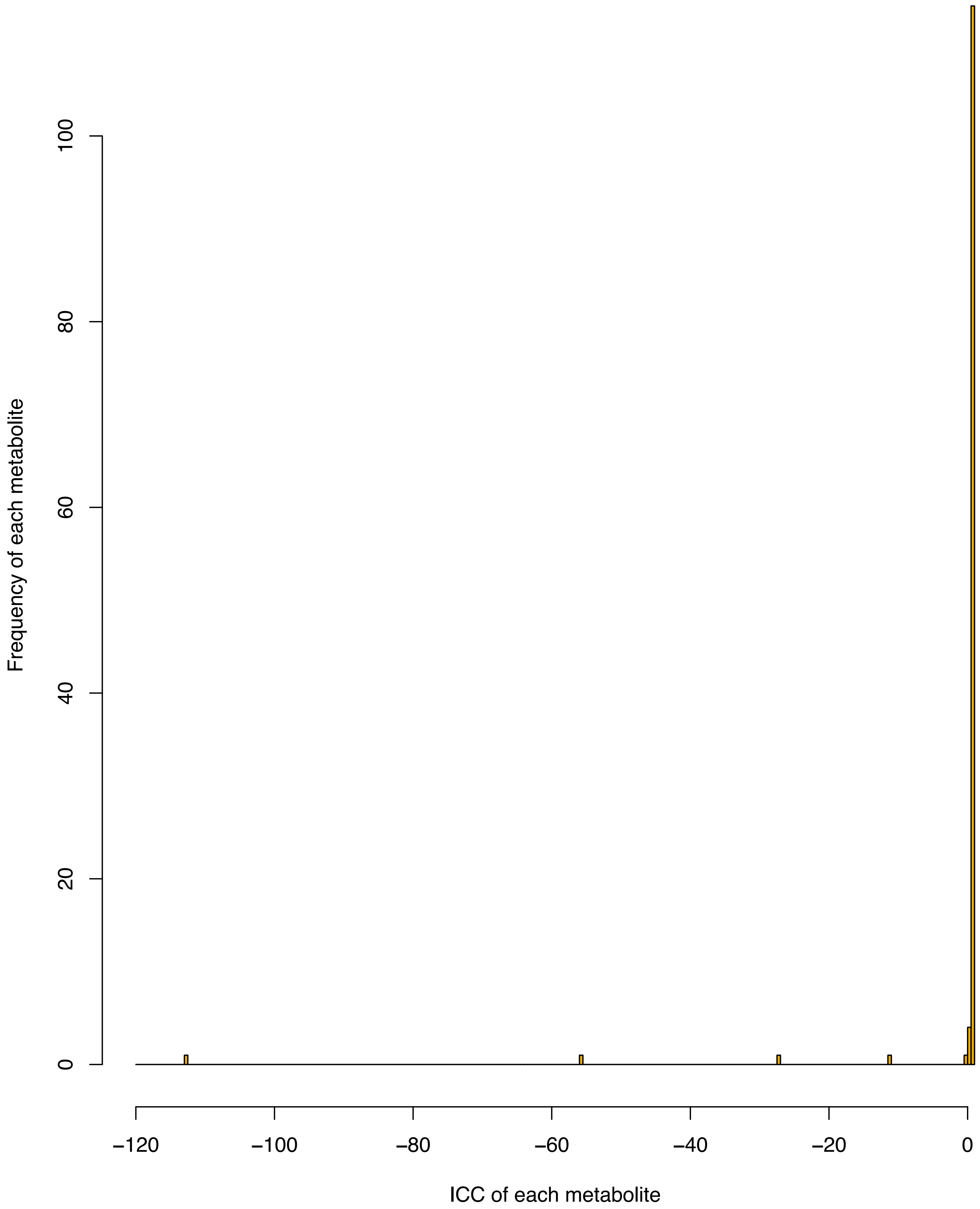


R Core Team (2018). R: A language and environment for statistical computing. R Foundation for Statistical Computing, Vienna, Austria. URL https://www.R-project.org/.
